# Detecting Zn(II) Ions in Live Cells with Near-Infrared Fluorescent Probes

**DOI:** 10.3390/molecules24081592

**Published:** 2019-04-22

**Authors:** Mingxi Fang, Shuai Xia, Jianheng Bi, Travis P. Wigstrom, Loredana Valenzano, Jianbo Wang, Marina Tanasova, Rudy L. Luck, Haiying Liu

**Affiliations:** 1 Department of Chemistry, Michigan Technological University, 1400 Townsend Drive, Houghton, MI 49931, USA; mfang@mtu.edu (M.F.); shuaix@mtu.edu (S.X.); jbi1@mtu.edu (J.B.); tpwigstr@mtu.edu (T.P.W.); lvalenza@mtu.edu (L.V.); mtanasov@mtu.edu (M.T.); 2 College of Biological, Chemical Sciences and Engineering, Jiaxing University, Jiaxing 314001, China

**Keywords:** near-infrared fluorescence, fluorescent probes, Zn(II), di-(2-picolyl)amine, living cells, cellular imaging

## Abstract

Two near-infrared fluorescent probes (**A** and **B**) containing hemicyanine structures appended to dipicolylamine (DPA), and a dipicolylamine derivative where one pyridine was substituted with pyrazine, respectively, were synthesized and tested for the identification of Zn(II) ions in live cells. In both probes, an acetyl group is attached to the phenolic oxygen atom of the hemicyanine platform to decrease the probe fluorescence background. Probe **A** displays sensitive fluorescence responses and binds preferentially to Zn(II) ions over other metal ions such as Cd^2+^ ions with a low detection limit of 0.45 nM. In contrast, the emission spectra of probe **B** is not significantly affected if Zn(II) ions are added. Probe **A** possesses excellent membrane permeability and low cytotoxicity, allowing for sensitive imaging of both exogenously supplemented Zn(II) ions in live cells, and endogenously releases Zn(II) ions in cells after treatment of 2,2-dithiodipyridine.

## 1. Introduction

After iron, zinc is the most abundant metal ion in the human body. Zn occurs strongly bonded within metalloproteins providing structural support and accomplishing various catalytic functions [1,2,3,4,5,6,7]. Zn(II) is essential for biological processes including signal transmission, gene expression, cellular metabolism, brain function, apoptosis, metalloenzyme regulation, neurotransmission, and mammalian reproduction [1,2,3,4,5,6,7]. Malfunctioning Zn(II) homeostasis can result in various diseases in the human body ranging from Alzheimer’s disease to cancers and infantile diarrhea [1,8,9]. Therefore, accurate quantification and imaging of Zn(II) ions are vital for understanding their function in biological, physiological, and pathological processes [1]. The detection of Zn(II) ions at 10^−4^ M concentration in some vesicles and 10^−10^ M concentration in the cytoplasm has been reported [1,10,11,12,13,14]. Unfortunately, many of the reported probes suffer from interferences such as the water Raman peak, metal ions such as Cd^2+^ ions, autofluorescence from biological samples, and photo-damage to cells and tissues because the excitation wavelength is usually less than 600 nm. Fluorescent probes have been devised to address most of these issues and some offer deep tissue penetration in vivo imaging application. However, only a few near-infrared (NIR) fluorescent probes were reported to achieve sensitive visualization of Zn(II) in live cells [15,16,17].

**Scheme 1:**
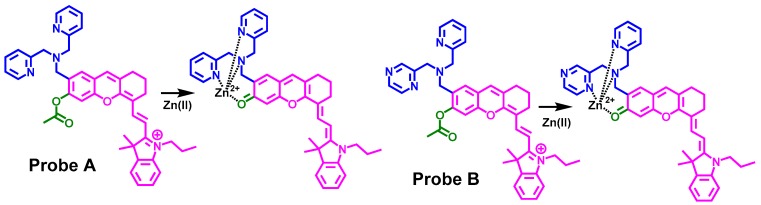
Drawings of the fluorescent probes and their interactions with Zn(II) ions.

**Scheme 2:**
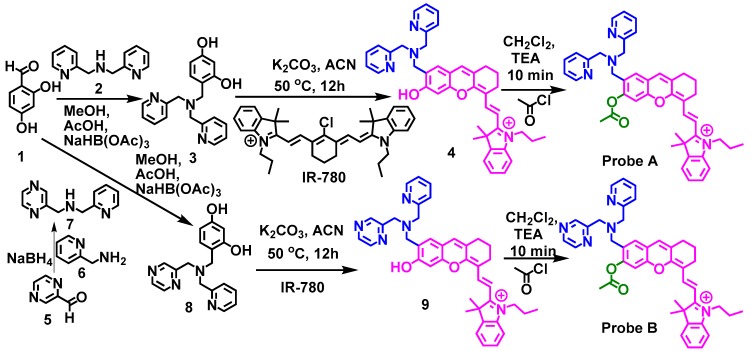
Synthetic approaches to prepare fluorescent probes **A** and **B**.

In this study, we report on the syntheses and properties of fluorescent probes for sensitive identification of Zn(II), see Scheme 1. These probes emit in the near-infrared region and they also possess excellent photostability and high molar absorptivity [18,19]. We outline the syntheses of probes **A** and **B**, see Scheme 1 and Scheme 2, and utilize these to determine Zn(II) concentrations in live cells by coordinating different Zn(II)-binding ligands on the hemicyanine platform. We find a significant reduction in the fluorescence background of the probes if an acetyl group is attached to the hydroxyl group on the hemicyanine platform. This may be due to less delocalization of the lone pair from the oxygen atom directly bonded to the acyl/hemicyanine fluorophore resulting in less charge delocalization via resonance as if there was only a hydroxyl group. The acetyl group is very stable to endogenous esterases and acetylated probes display an extremely weak and imperceptible fluorescence background in live cells that do not contain free Zn(II) ions [20]. However when present, Zn(II) ions coordinate with probe **A** and promote acetyl hydrolysis that yields a significant increase in the fluorescence of the hemicyanine fluorophore in both solution and live cells. We demonstrate that probe **A** can reversibly monitor Zn(II) ion concentrations in live cells and allows for sensitive detection of free endogenous Zn(II) released after 2,2-dithiodipyridine is added to cells. In contrast, probe **B** displays a much smaller fluorescence increase with the addition of Zn (II) ions.

## 2. Experimental Section

### 2.1. Instrumentation

Details of equipment utilized for NMR and mass spectrometry are as previously reported [21]. Standard 1 cm path length quartz fluorescence cuvettes were used to collect all absorption and emission spectra at room temperature. Absorbance spectra were obtained on a Perkin Elmer Lambda 35 UV/Vis Spectrometer (PerkinElmer, Inc., Maltham, MA, USA). The fluorescence spectra were obtained on a Horiba (Jobin Yvon, Edison, NJ, USA) Fluoromax-4 Spectrofluorometer equipped with a 150 W CW ozone-free xenon arc lamp. A buffer of 10 mM HEPES (pH = 7.0) was used to measure the sensing performance of Zn(II) and for the selectivity measurements of the probe. ZnCl_2_ was used as the source of Zn(II) for all optical measurements. For each experiment, zinc chloride stock solutions (10 mM) were prepared freshly. In the selectivity measurements, individual solutions of different metal ions were added to solutions containing 5 μM of either probe A or B in an EtOH/buffer (1/99, *v/v*) co-solvent system. Subsequently, a different volume of the zinc stock solution was added to each solution and then thoroughly mixed. The absorption and fluorescence spectra were obtained 10 min after the addition of Zn(II) ions. Both the excitation and emission slit widths were set to 5 nm and fluorescence spectra were obtained at excitation of 640 nm.

In order to explain and confirm the excitation patterns observed experimentally for intermediates **4** and **9**, see Scheme 2, probes **A** and **B** with and without the presence of a [Zn (OH)]^−^ moiety, the density functional theory (DFT) was employed as implemented in the Gaussian09 program package [22]. The solvated environment of aqueous buffer was mimicked via the CPCM continuum model. Structures were first allowed to geometrically affirm at the PBE/6-311G(d,p) level of theory which allowed the HOMO/LUMO energy gaps to be calculated at the PBE/6-311++G(d,p) level of theory. The time-dependent density functional theory (TDDFT) was employed at the level of theory mentioned above. For the initial calculations, three excited states were explored for all the above molecular models investigated. In addition, we conducted additional modeling with [Zn(OH_2_)]^2+^ and [Zn(OH_2_)_2_]^2+^ moieties attached to probes **A** and **B**. These were conducted with the APFD functional [23] and the 6-311+G(2d,p) basis set for the TDDFT calculation using the Gaussian16 program package [24]. Full details of the calculations are presented in the Supplementary Materials.

### 2.2. Cell Culture and Fluorescence Imaging

HeLa cells were obtained and prepared for confocal imaging as previously reported [25,26]. This pertains to their initial incubation, addition of either probe, addition of fresh serum free media, and final washings with PBS [15]. The live cell images were taken by a confocal fluorescence microscope (Olympus IX 81). The fluorescence images were obtained at 60× magnification and the laser energies were kept constant for each image series. The red channel fluorescence of the probes was excited at a wavelength of 635 nm and fluorescence was collected from 650–750 nm.

For the Zn(II) chelate test, after adding the corresponding concentration of Zn(II) plus sodium pyrithione (Pyr), 100 μM of TPEN (*N,N,N’,N’*-tetrakis(2-pyridylmethyl)ethylenediamine) was added to each confocal dish, and the cells were incubated for 10 min at room temperature. The intracellular level of Zn(II) ions in HeLa cells was evaluated using 2,2’-dithiodipyridine (DTDP). The cells were serum starved for 3 h at 37 ℃ with 5% CO_2_ and then incubated with 1 μM of probe **A** for 30 min. Subsequently, 100 μM DTDP was added and cells were further incubated for 30 min at 37 ℃ with 5% CO_2_. Then, 100 μM TPEN (*N,N,N,N*-tetrakis(2-pyridinylmethyl)-1,2-ethanediamine) was added and the mixture incubated for 10 min at room temperature. Cell images were taken by a confocal fluorescence microscope (Olympus IX 81, Olympus America Inc., Nelville, NY, USA). The excitation wavelength was 635 nm and the images were collected at 675–725 nm. The cytotoxicity of the probes was determined using an MTS assay as previously reported [25,26].

### 2.3. Materials

Unless indicated, all reagents and solvents were obtained from commercial suppliers and used without further purification.

Synthesis of compound **3**: 2,4-Dihydroxybenzaldehyde (1.086 g, 7.9 mmol) and di-(2-picolyl)amine (1.57g, 7.9 mmol) were dissolved in 30 mL methanol in a 100-mL round-bottom flask. Acetic acid (5 mL) and sodium triacetoxyborohydride (1.5 g, 7 mmol) were added and the reaction mixture stirred at room temperature for 3 days. After the starting materials were depleted (verified by TLC monitoring), dilute HCl solution was added to quench the unreacted sodium salt, and methanol was removed under vacuum. The pH of the reaction mixture was adjusted to 9.0. The resulting crude was extracted with ethyl acetate and washed with water three times, followed by drying over Na_2_SO_4_. The residue was collected by removing the solvent under vacuum and purified by recrystallization with diethyl ether to afford the product **3**, Scheme 2, in the form of a yellow flaky solid (1.2 g, 47%).

Synthesis of compound **4**: Compound **3**, Scheme 2, (302 mg, 0.94 mmol) and K_2_CO_3_ (130 mg, 0.94 mmol) were dissolved in acetonitrile (10 mL) in a 50 mL round-bottom flask. After the mixture was stirred at room temperature under a N_2_ atmosphere for 15 min, a solution of IR-780 iodide (314 mg, 0.47 mmol) in CH_3_CN (2.5 mL) was added to the mixture via a syringe, and the mixture was heated to 50 °C for 5 h. When the solvent was removed under reduced pressure, the crude product was purified by silica gel chromatography using CH_2_Cl_2_/MeOH (15:1, *v/v*) as eluent, affording compound 4 as an aquamarine solid (160 mg, 55%). ^1^H NMR (400 MHz, CDCl_3_): δ 8.57–8.50 (m, 2H), 7.64–7.61 (m, 1H), 7.42–7.38 (m, 2H), 7.31–7.27 (m, 3H), 7.17–7.14 (m, 2H), 7.10–7.03 (m, 3H), 6.86 (s, 1H), 6.67–6.63 (m, 2H), 6.34–6.29 (m, 1H), 4.26 (s, 2H), 3.87–3.67 (m, 6H), 2.65 (s, 4H), 1.89–1.56 (m, 10H), 1.04–0.97 (s, 3H); ^13^C NMR (100 MHz, CDCl_3_) δ 176.1, 163.4, 162.9, 157.9, 155.0, 149.4, 148.6, 145.0, 142.0, 141.5, 137.6, 136.1, 130.0, 129.3, 126.8, 126.3, 123.8, 123.4, 122.8, 122.5, 115.2, 114.7, 112.4, 103.5, 102.7, 58.6, 55.9, 53.7, 50.3, 47.2, 29.2, 28.6, 28.0, 24.7, 21.4, 20.6, 11.9. HRMS (ESI): calculated for C_41_H_43_N_4_O_2_^+^ [M]^+^: 623.3381; found: 623.3629.

Synthesis of fluorescent probe **A**: Compound **4**, Scheme 2, (100 mg, 0.13 mmol) was added to a solution of acetyl chloride (0.05 mL, 0.26 mmol) and triethylamine (0.1 mL) in anhydrous dichloromethane (10 mL) and stirred for 15 min under a nitrogen atmosphere. The solvent was removed under reduced pressure yielding a blue crude product which was washed with water and brine thrice. The product was dissolved in dichloromethane and dried over Na_2_SO_4_ and subsequently filtered. The filtrate was collected and the solvent removed affording probe A. ^1^H NMR (400 MHz, CDCl_3_): δ 8.59–8.52 (m, 3H), 7.72–7.63 (m, 2H), 7.51–7.45 (m, 3H), 7.31 (s, 1H), 7.22–7.15 (m, 3H), 7.00–6.93 (m, 3H), 6.82 (s, 1H), 6.68–6.64 (m, 1H), 4.74–4.53 (m, 2H), 3.91–3.63 (m, 6H), 2.71 (s, 4H), 2.31–2.20(m, 3H), 1.90–1.33 (m, 10H), 1.22 (s, 3H); ^13^C NMR (100 MHz, CDCl3) δ 178.6, 169.2, 160.3, 152.2, 151.3, 150.1, 148.9, 148.7, 146.4, 142.2, 141.6, 137.2, 137.1, 132.3, 132.2, 130.4, 129.6, 129.5, 129.2, 128.7, 128.4, 128.1, 123.6, 122.8, 122.6, 120.2, 115.7, 113.6, 110.3, 106.6, 60.0, 54.1, 52.3, 51.2, 48.2, 32.1, 29.9, 28.5, 24.7, 22.9, 21.8, 21.3, 11.9. HRMS (ESI): calculated for C_43_H_45_N_4_O_3_^+^ [M]^+^: 665.3486; found: 665.3474.

Synthesis of compound **7**: Pyrazine-2-carbaldehyde (1 g, 9.25 mmol) was dissolved in methanol (90 mL) and yridine-2-methylanamine (0.96 mL, 9.25 mmol) was added by a syringe. This mixture was stirred at 25 °C for 30 min followed by rapid addition of NaBH_4_ (1.06 g, 27.76 mmol), and then stirred at 25 °C for an additional 2 h. The solvent was evaporated under reduced pressure, water (20 mL) was added to the residue and the solution pH was adjusted to approximately 10 by adding Na_2_CO_3_ solution. The mixture was extracted with CH_2_Cl_2_ (3×30 mL) and purified by column chromatography with CH_2_Cl_2_/MeOH (30:1, *v/v*) to give the product **7**, Scheme 2, as a pale-yellow oil (1.6 g, 90% yield).

Synthesis of compound **8**: Compound **8**, Scheme 2, was prepared using compound **7** (0.9 g, 4.5 mmol) and 2,4-dihydroxybenzaldehyde (0.68 g, 5 mmol) according to the method used for compound **3**, affording the product as a yellow flaky solid (0.7 g, 44%). ^1^H NMR (400 MHz, CDCl_3_): δ 8.54 (d, *J* = 1.6 Hz, 1H), 8.50–8.49 (m, 1H), 8.46–8.45 (m, 1H), 8.36 (d, *J* = 2.4 Hz, 1H), 7.60–7.56 (m, 1H), 7.27 (d, *J* = 7.6 Hz, 1H), 7.14–7.11 (m, 1H), 6.81 (d, *J* = 8.4 Hz, 1H), 6.40 (d, *J* = 2.4 Hz, 1H), 6.27–6.24 (m, 1H), 3.87 (s, 4H), 3.66 (s, 2H); ^13^C NMR (100 MHz, CDCl3) δ 158.4, 158.3, 157.8, 154.5, 148.8, 145.3, 144.0, 143.2, 137.5, 131.1, 123.6, 122.8, 114.2, 106.9, 104.2, 58.9, 56.8. HRMS (ESI): calculated for C_18_H_19_N_4_O_2_^+^ [M + H]^+^: 323.1530; found: 323.1495.

Synthesis of compound **9**: Compound **9**, Scheme 2, was carried out using compound **8** (160 mg, 0.25 mmol) and IR-780 (330 mg, 0.49 mmol) in a similar manner to that used for compound **4**, affording 130 mg product (46% yield). ^1^H NMR (400 MHz, CDCl_3_): δ 8.62–8.42 (m, 7H), 7.69 (s, 1H), 7.43–7.29 (m, 6H), 7.04 (s, 1H), 6.44 (s, 1H), 4.38 (s, 2H), 3.98–3.90 (m, 6H), 2.75 (s, 4H), 1.92–1.76 (m, 10H), 1.23 (s, 3H); ^13^C NMR (100 MHz, CDCl3) δ 176.5, 158.7, 157.8, 157.1, 155.0, 154.4, 154.1, 149.1, 148.7, 145.5, 143.9, 143.3, 142.0, 141.6, 137.8, 137.2, 135.5, 131.1, 129.8, 129.3, 126.9, 123.4, 122.7, 115.4, 114.6, 112.6, 106.6, 104.1, 58.9, 56.8, 56.1, 50.5, 47.5, 29.3, 28.6, 24.9, 21.8, 21.4, 20.6, 11.9. HRMS (ESI): calculated for C_40_H_42_N_5_O_2_^+^ [M]^+^: 624.3333; found: 624.3314.

Synthesis of fluorescent probe **B**: Probe **B**, Scheme 2, was carried out by reacting compound **9** with acetyl chloride in a similar manner to that for probe **A**. ^1^H NMR (400 MHz, CDCl3): δ 8.71–8.45 (m, 7H), 7.68–7.67 (m, 1H), 7.51–7.41 (m, 6H), 7.11 (s, 1H), 6.97 (s, 1H), 4.73 (s, 2H), 3.87–3.71 (m, 6H), 2.86–2.70 (m, 4H), 2.30 (s, 3H), 2.02–1.77 (m, 10H), 1.23 (s, 3H); ^13^C NMR (100 MHz, CDCl3) δ 178.7, 169.1, 168.8, 152.3, 151.2, 150.8, 146.5, 145.5, 145.4, 144.2, 144.1, 143.6, 142.3, 141.6, 141.5, 132.7, 131.1, 130.6, 129.5, 129.0, 128.1, 122.7, 122.5, 120.4, 120.3, 115.9, 113.5, 110.3, 68.4, 57.9, 52.7, 51.2, 47.9, 32.1, 30.6, 29.9, 28.4, 24.7, 21.3, 20.5, 11.8. HRMS (ESI): calculated for C_42_H_44_N_5_O_3_^+^ [M]^+^: 666.3439; found: 666.3436.

## 3. Results and Discussion

### 3.1. Probe Design and Synthesis

The synthetic route to prepare probes **A** and **B** is outlined in Scheme 2. In order to bind dipicolylamine (DPA) (a ligand known to bind Zn(II) ions) to the hemicyanine dye, we prepared 4-((bis(yridine-2-ylmethyl)amino)methyl)benzene-1,3-diol (**3**) by first reacting 2,4-dihydroxybenzaldehyde with DPA and then reducing the enamine intermediate with sodium triacetoxyborohydride. Compound **3** was reacted with cyanine dye IR-780 (a chloro-substituted tricarbocyanine dye) under basic conditions in acetonitrile, to afford the hemicyanine dye bearing the dipicolylamine residue (**4**). An acetyl group was then bonded to the hydroxyl moiety on the hemicyanine fluorophore to significantly reduce the probe fluorescence yielding fluorescent probe **A**. Probe **B** was prepared in a similar manner to that of probe **A** except that the compound 1-(pyrazin-2-yl)-N-(yridine-2-ylmethyl)methanamine, **7**, prepared by reacting compounds **5** with **6**, see Scheme 2, was utilized. This should result in weaker binding for the Zn(II) ions due to reduced basicity and potentially a reduction in probe fluorescence.

### 3.2. Optical Properties of Fluorescent Probes **A** and **B** in Different Solvents

The absorption spectra of intermediates **4** and **9**, and probes **A** and **B** were investigated in ethanol, tetrahydrofuran (THF), and buffer (pH 7.0) containing 1% ethanol, see Table 1. Intermediate **4** displays absorption peaks at 708 nm, 705 nm and 687 nm, and fluorescence peaks at 725 nm, 718 nm and 703 nm in ethanol, tetrahydrofuran (THF), and buffer (pH 7.0) containing 1% ethanol at 635 nm excitation, respectively. Increasing solution polarity results in blue shifts of absorption and fluorescence peaks for intermediate **4** and causes fluorescence quenching as intermediate **4** has the lowest fluorescence quantum yield of 1.4% in buffer (pH 7.0) containing 1% ethanol. This may be due to fluorescence quenching photo-induced electron transfer from the tertiary amine of the Zn(II)-binding dipicolylamine residue to the hemicyanine fluorophore. The fluorescent probe A displays lower fluorescence quantum yields than intermediate **4** and this may be due to the presence of the acetyl group attached to the phenolic oxygen atom of the hemicyanine platform in probe **A**. This significantly reduces the electron donating ability of the phenolic oxygen atom and decreases fluorescence. Similar results to intermediate **4** and probe **A** were observed with intermediate **9** and probe **B**.

### 3.3. Absorbance Responses of Intermediates and the Probes to Zn(II) Ions

**Figure 1:**
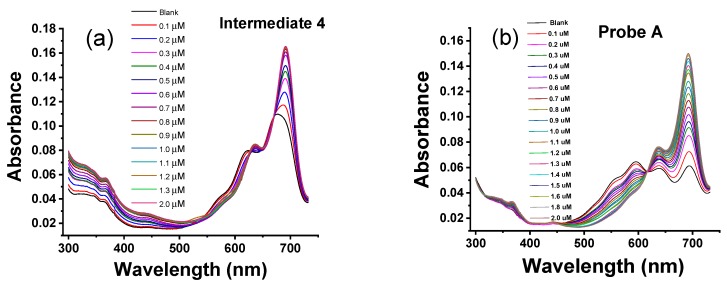
(**a**) UV-Vis absorption spectra of 1.0 µM intermediate **4** and (**b**) UV-Vis absorption spectra of probe **A** upon gradual addition of Zn(II) from 0.1 µM to 2.0 µM to 10 mM HEPES buffer solutions (pH 7.0) containing 1.0 μM probe **A**.

**Figure 2:**
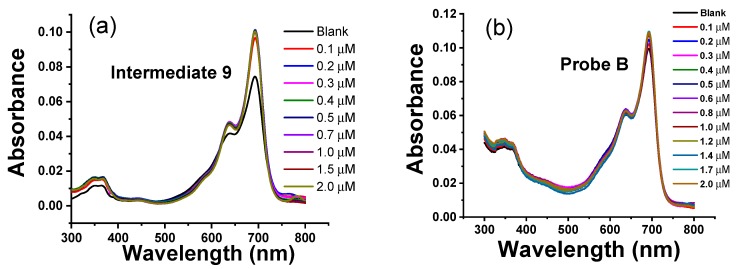
(**a**) UV-Vis absorption spectra of 1.0 µM intermediate **9** and (**b**) UV-Vis absorption spectra of 1.0 µM probe **B** upon gradual addition of Zn(II) from 0.1 µM to 2.0 µM to 10 mM HEPES buffer solutions (pH 7.0) containing 1.0 μM) probe **B**.

The absorption responses of intermediates **4** and **9**, and fluorescent probes **A** and **B** to Zn(II) ions were evaluated in aqueous HEPES buffer (pH 7.0) containing 1% ethanol (Figure 1). Intermediate 4 contains a main absorption peak at 691 nm and a shoulder peak at 636 nm (Figure 1). The addition of Zn(II) ions from 0.1 µM to 2.0 µM results in gradual increases of the main absorption peak at 691 nm (Figure 1). Probe **A** at the same concentration shows a lower absorbance than intermediate **4** due to the presence of the acetyl group and for reasons discussed above. However, the addition of Zn(II) ions from 0.1 µM to 2.0 µM significantly increases the main absorption peak at 691 nm with a more moderate increase in the shoulder peak at 636 nm (Figure 1). This is because Zn(II) facilitates hydrolysis of the acetyl group and affords rigidity to the overall structure facilitating the absorption. Interestingly, both intermediate **9** and probe **B** are not as sensitive to increasing concentrations of Zn(II) ions, as addition of up to 20 µM concentration only results in slight increases in both the main absorption and the shoulder peaks at 691 nm and 636 nm, respectively (Figure 2).

### 3.4. Fluorescence Response of the Intermediates and Probes to Zn(II) Ions

**Figure 3:**
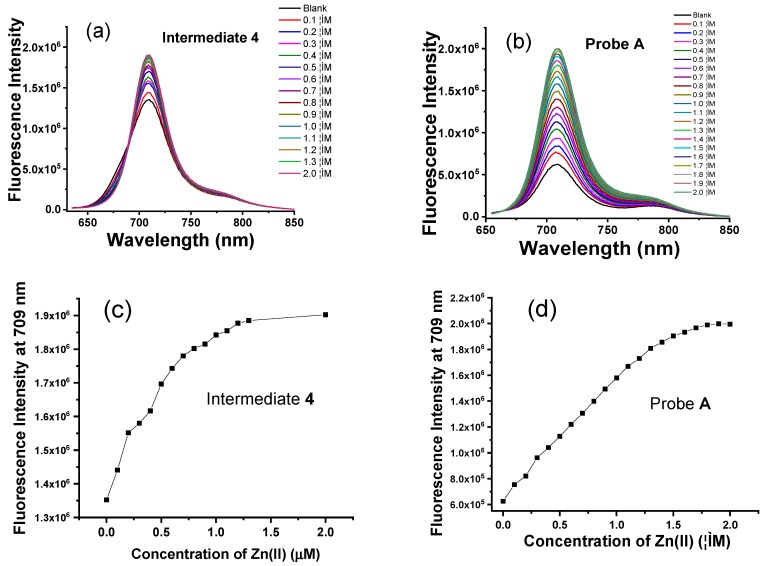
(**a**) Fluorescence spectra of intermediate **4**, and (**b**) probe **A** at 635 nm excitation upon gradual addition of Zn(II) from 0.1 µM to 2.0 µM to 10 mM HEPES buffer solutions (pH 7.0) containing 1.0 μM probe **A**. (**c**) Intensity dependence on zinc ion concentration of 1.0 µM intermediate **4** and (**d**) probe **A** upon gradual addition of Zn(II) from 0.1 µM to 2.0 µM to 10 mM HEPES buffer solutions (pH 7.0) containing 1.0 μM probe **A.**

**Figure 4:**
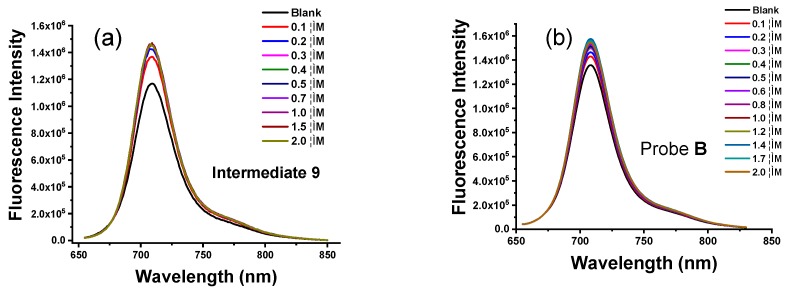
(**a**) Fluorescence spectra of 1.0 µM intermediate **9** and (**b**) probe **B** at 635 nm excitation upon gradual addition of Zn(II) from 0.1 µM to 2.0 µM to 10 mM HEPES buffer solutions (pH 7.0) containing 1.0 μM probe **B**.

Intermediate **4** displays strong fluorescence in the absence of Zn(II) ions, indicating that the photo-induced electron transfer from the tertiary amine of the dipicolylamine residue to the hemicyanine fluorophores is unable to completely quench the fluorescence of the hemicyanine platform (Figure 2). The addition of Zn(II) ions, up to 2.0 µM, cause a moderate increase in the fluorescence peak at 708 nm, see Figure 3. However, fluorescent probe **A** exhibits a much weaker fluorescence in the absence of Zn(II) ions and its fluorescence dramatically increases upon the addition of Zn(II) ions from 0.1 µM to 2.0 µM (Figure 2). This sensitive response of probe **A** to Zn(II) ions arises from the quick hydrolysis of the acetyl group via binding of Zn(II) to the dipicolylamine residue. Probe **A** displays a linear fluorescence response to Zn(II) from 0.1 µM to 1.5 µM with a detection limit of 0.45 nM, see Figure S8. The titration curve fitted well up to a 1:1 stoichiometry and the Hill plot displays a linear relationship with a slope of 1.0 and the Job’s plot contains a maximum point at a mole fraction of 0.50, see Figure S11. Probe **A** shows high affinity for Zn(II) ions with a lower dissociation constant of 9.5 × 10^5^ M^−1^ as determined by a fluorescence titration curve, see Figure S7. In contrast, intermediate **9** and probe **B** display slight increases in fluorescence if up to 10 times higher concentration of Zn(II) ions is added to their solutions (Figure 4). This difference may be ascribed to the weaker binding of Zn(II) ions as a result of the substitution of one pyridine by pyrazine.

The accuracy of the fluorescence response for probe **A** was assessed as greater than 92%. Concentrations of Zn^2+^ were accurately determined using a calibration curve based on atomic absorption, Figure S30, and compared to fluorescence measurements using probe **A**, Figure S32.

### 3.5. Selectivity Studies

The selectivity of fluorescent probes **A** and **B** to Zn(II) over other metal ions was evaluated in a 10 mM HEPES buffer (pH 7.0). No change in fluorescence was observed with probe **A** if up to 20 µM alkali and alkaline-earth metal ions, specifically Na^+^, K^+^, Ca^2+^, and Mg^2+^ ions, and some transition metal ions, i.e., Mn^2+^, Fe^2+^, Ni^2+^, Cu^2+^, Cd^2+^, and Hg^2+^ ions were added (Figure S9(a)). In contrast probe **B** did not display selectivity for Zn)II in a similar comparison (Figure S9(b)). Probe **A** shows high selectivity to Zn(II) ions over other metal ions in contrast to the other reported fluorescent probes for detection of Zn(II) ions which show significant interference from Cd^2+^ ions.

### 3.6. Photostability of the Probes

The photostability of the probes was evaluated by comparative assessment of fluorescent intensity every 10 min under continuous excitation at 635 nm using a 150 W xenon arc lamp. Probe **A** displays excellent photostability as its fluorescence intensity decreases by only 3.0% after one hour of excitation and by 7.6% after three hours excitation (Figure S10). Probe **B** exhibits similar photostability to probe **A**. Fluorescent intensity losses of 4.2% after one hour of excitation and 7.0% under three hours of excitation are obtained (Figure S10).

### 3.7. Theoretical Modeling Results

Analysis of the molecular charges allowed for appreciating changes in charge distribution with the PBE functional calculations. The distal nitrogen atom in the pyrazine moiety (i.e., that which binds to the Zn atom) has a notable charge change (+0.049|e|) when comparing pyrazine in probe **B** to the N atom in pyridine in probe **A**, which is in agreement with the known lesser basicity for pyrazine. This change in charge results in an increase in the bond length of 0.046 Å in the N-Zn bond (now 2.0995 Å), indicating a decrease in bond strength. This weaker coordination of the Zn(II) ion may be responsible for the subdued increase in fluorescence when pyrazine is present (probe **B**) in place of pyridine (probe **A**).

The time dependent excitation patterns for all the compounds investigated is reported in Table S9. The results for the probes, including the presence of an additional ligand (i.e., OH^−^), are summarized in Table S12. The absorption peaks computed for intermediate **4**, intermediate **9**, and probes **A** and **B** are 699 nm, 701 nm, 693nm, and 699 nm, respectively, and they are in excellent agreement with experimental evidence (see Table S10). When Zn(II) is added to probe **A**, a slight shift in the absorption peak (~+40 nm) is observed. Interestingly, visual inspection of the molecular orbitals indicates that such a value corresponds to an electron excitation from the HOMO to the LUMO, instead of the HOMO-1/LUMO transition observed for the other molecules calculated (see Table S11). The overestimation of the excitation energy may be related to a deficiency in the PBE function in properly handling the delocalization of the electronic charge of the nitrogen-rich molecular moiety when forming the Zn complex.

However, the result computed for the binding of probe **B** to the Zn atom is in excellent agreement with experimental results with a calculated absorption peak of 702 nm. It is also ascribed to the excitation that occurs from the HOMO-1 to the LUMO. The lobes for the HOMO orbital for probe **A**-Zn(OH) indicate a substantial contribution from the lone pairs on the tertiary amine on the dipicolylamine moiety in contrast to a lack of such overlap in the HOMO-1 orbital for probe **B**-Zn(OH). Additionally, while Zn complex **4**, see Table S11, has a HOMO/LUMO transition, delocalization into the tertiary N atom is not observed, indicating that completing the coordination sphere around Zn is required for this to occur. An exploration of the geometrical features of the Zn first coordination sphere reveals that the Zn atom coordinates with the available nitrogen and oxygen atoms of the probes. While the average atomic distances are not affected, some individual values do change, in particular, the Zn-N atom distance for the pyridine and pyrazine rings shows an increase of approximately 0.05 Å from probe **A** to probe **B**. Such a structural change is reflected by a decrease in the negative charge of the nitrogen of approximately 0.07 |e|. This observation seems to confirm a limitation in the chosen DFT function in dealing with the Zn complex arising from probe **A**. It is interesting to note that results obtained in the presence of the counter anion, confirm the observations reported above with absorption peaks for Probe **A-Zn** and Probe **B-Zn** of 765 nm and 711 nm, respectively.

The coordination sphere bond distance results from the additional calculations, i.e., with the APFD/6-311+G(2d,p) functional/basis set combination, which consisted of the [Zn(OH_2_)]^2+^ and [Zn(OH_2_)_2_]^2+^ moieties attached to probes **A** and **B**, are illustrated in Figure S29, (see supporting information pages 11–35. As is evident, trigonal bipyramidal and octahedral geometries are obtained with these various entities and there is crystallographic evidence of the former mode of coordination. The fact that pyrazole is a weaker ligand is again confirmed by the longer Zn-N (pz) distances obtained with both probes, i.e., 2.075 Zn(OH_2_)^2+^ and 2.120 [Zn(OH_2_)_2_]^2+^ compared to 2.049 and 2.091 Å for probes **B** and **A**, respectively. In these structures the phenolic C-O distances were equivalent, leading to the conclusion that the conjugation and fluorescence intensities for the hemicyanine platform should be equivalent. This is also evident in the calculated excited state transitions which consisted of the HOMO to LUMO transition in all cases, see Table S12. It is noteworthy that this calculation strategy, i.e., APFD/6-311+G(2d,p), resulted in values for the transitions that were ~140 nm less than those observed experimentally (and at 0.426 and 0.412 eV for probes **A** and **B**, respectively, and were greater than the expected error range of 0.20–0.25 eV [23]) in contrast to the excellent agreement noted above for PBE/6-311++G(d,p).

### 3.8. Cytotoxicity of the Probes

**Figure 5:**
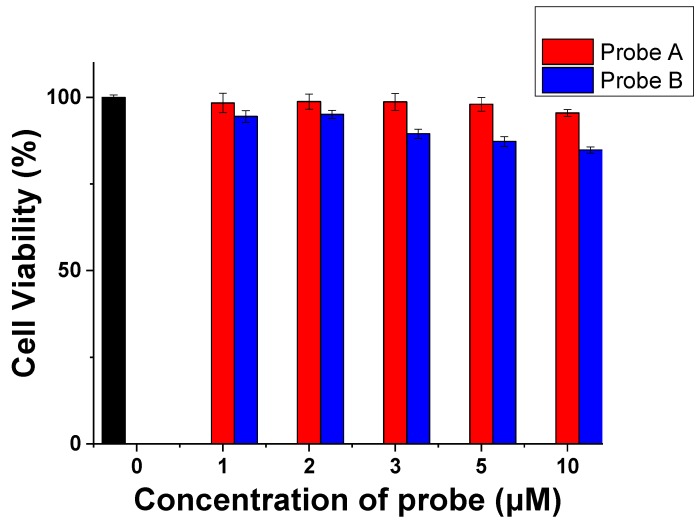
Cytotoxicity and cell proliferation of probes **A** and **B** conducted by MTS assay. HeLa cells were incubated with 1, 2, 3, 5, and 10 µM of probes for 48 h, and cell viability was measured by adding MTS reagent and measuring at 490 nm at the average of three times. The black bar represents a blank control.

The cytotoxicity of probes **A** and **B** was determined by carrying out the MTS assay (Figure 5). Incubation of the HeLa cells with the probes did not have any significant impact on cell viability even at 10 µM concentration. Overall, the probe showed insignificant cytotoxicity over 48 h at the test concentrations, with cell viability of 95%, indicating that the probes have good biocompatibility and can serve as an excellent staining reagent for live cell.

### 3.9. Live Cell Imaging of Fluorescent Probes

**Figure 6:**
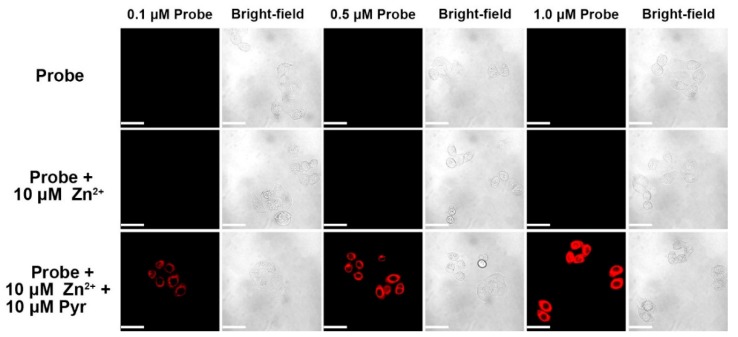
Fluorescence images of fluorescent probe **A** with concentration at 0.1 µM, 0.5 µM, and 1.0 µM in HeLa cells. Cells were incubated with probe **A** with specific concentration for 30 min. Cells were then supplemented with either 10 µM of zinc(II) chloride or 10 µM each of zinc(II) chloride plus sodium pyrithione (Pyr) for 30 min before acquiring images. Scale bar: 50 µm. λ_ex_: 635 nm.

In light of the aforementioned fluorescence analysis, we investigated only probe **A** to detect Zn(II) ions in live cells by incubating HeLa cells with different concentrations of probe **A** (0.1 µM, 0.5 µM, 1.0 µM) for 30 min at 37 °C prior to imaging. There was an extremely weak and barely perceptible fluorescence due to the low level of endogenous auto-fluorescence from the cells in the near-infrared region and the low fluorescence quantum yield of probe **A**, see Figure 6. This indicated that the ester bond of the probe was very stable inside live cells. The addition of 10 µM exogenous Zn(II) to the cells without the ionophore pyrithione did not cause a noticeable change in the fluorescence background. However, intracellular fluorescence increased significantly in response to exogenous Zn(II) in the presence of pyrithione. The cellular fluorescence intensity increased with an increase of the probe concentration from 0.1 µM to 1.0 µM.

**Figure 7:**
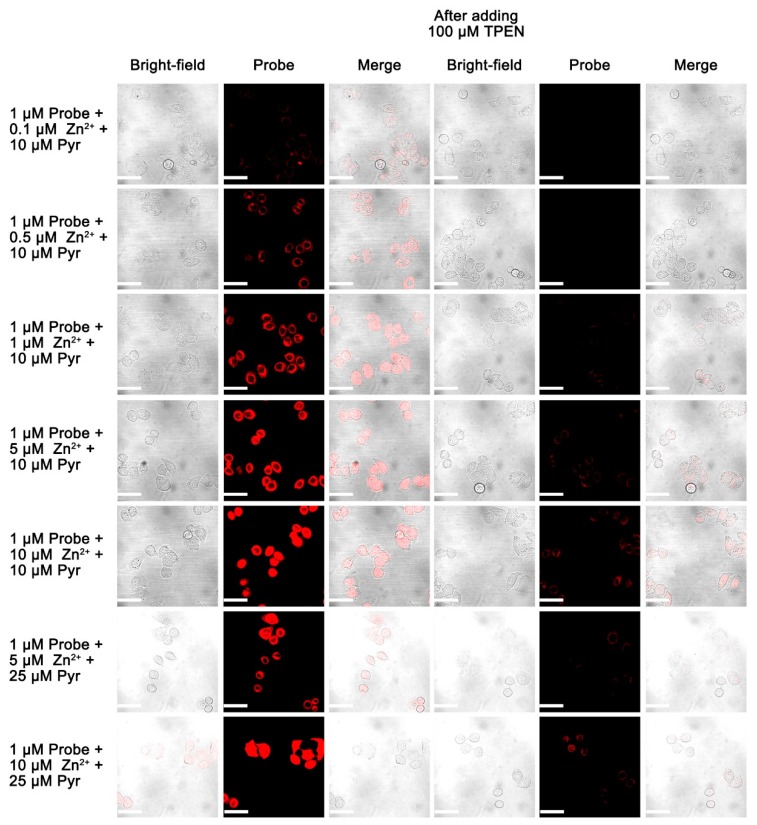
Fluorescence images of 1.0 µM fluorescent probe **A** in HeLa cells. Cells were incubated with probe **A** with specific concentration for 30 min. Cells were then supplemented with either zinc(II) chloride with different concentrations or zinc II) chloride with different concentrations plus sodium pyrithione (Pyr) for 30 mins before acquiring images. Then 100 µM TPEN (zinc chelator) was added for 10 min before acquiring the second set of images. Scale bar: 50 µm. λ_ex_: 635 nm.

The sensitivity of the probe to the intracellular Zn(II) level was also investigated by using a probe concentration of 1.0 µM, followed by stepwise increases in the concentration of the Zn(II)/ionophore pyrithione from 0.1 µM, 0.5 µM, 1.0 µM, 5 µM, and 10 µM, see Figure 7. The intracellular fluorescence increased with an increase of Zn(II) ion concentration from 0.1 to 5 µM, and it did not change significantly with further increases in the Zn(II) ion concentration. Clearly, probe **A** can sensitively detect intracellular Zn(II) ion with at least 0.1 µM concentration. The intracellular fluorescence decreased significantly after a cell-permeable Zn(II) ion chelator *N,N,N’,N’*-tetrakis(2-pyridylmethyl)ethylenediamine (TPEN) was added to the cells, [27] indicating that the probe responded to intracellular Zn(II) ions reversibly since TPEN effectively removed Zn(II) ions from the probe because of its much stronger binding affinity.

**Figure 8:**
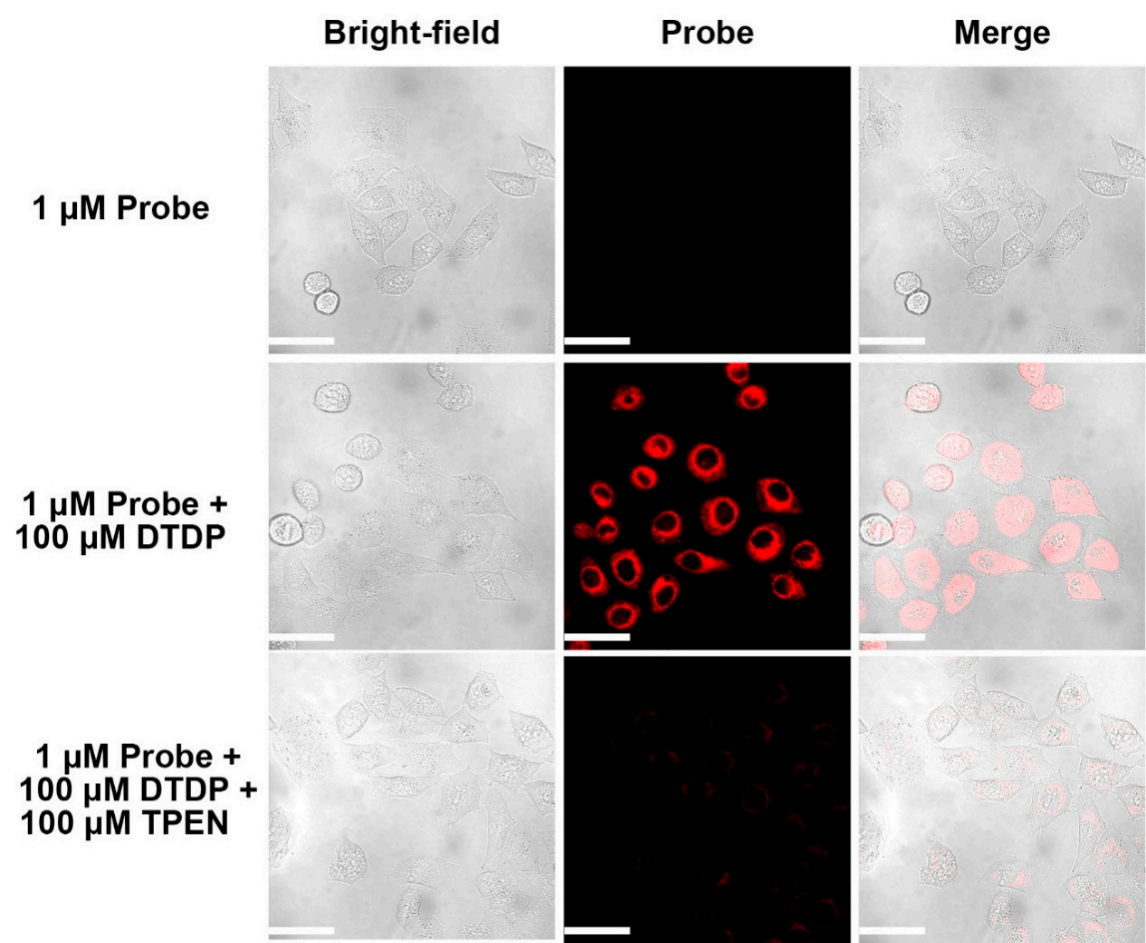
Fluorescence images of HeLa cells incubated with probe **A**. Cells were first incubated with 1.0 µM of probe **A** for 1 h and then were supplemented with 100 µM of 2,2’-dithiodipyridine (DTDP) for 30 mins before images were taken. Scale bar: 50 µm. λ_ex_: 635 nm.

In addition, we investigated the fluorescent probes to determine if they could be used to detect Zn(II) ions endogenously released from intracellular metalloproteins after the treatment with 2,2-dithiodipyridine (DTDP), see Figure 8. The DTDP treatment alone was reported to give modest increases of intracellular Zn(II) ions (nM) in cells. The probe fluorescence has imperceptible background in cells without DTDP treatment. However, a very strong fluorescence intensity from probe **A** (1.0 µA) was observed after DTDP treatment of HeLa cells without the addition of external ionophores, indicating the accumulation of endogenously released Zn(II) ions. The intracellular fluorescence was considerably and immediately reduced by TPEN treatment. These results clearly proved that fluorescent probe **A** sensitively detected endogenous Zn(II) ions in live cells.

## 4. Conclusions

We reported on the rational design, syntheses, and characterization of two novel near-infrared fluorescent probes for sensing Zn(II) ions based on the hemicyanine fluorophore. The fluorescent probe **A** containing the dipicolylamine residue is highly selective and sensitive to Zn(II) ions over other metal ions. The strong binding of Zn(II) ions to probe **A** facilitates the cleavage of the acetyl group and effectively increases the fluorescence of the fluorophore. The fluorescent probe **A** offers a way to live cell imaging of both exogenously supplemented Zn(II) ions and free endogenous Zn(II) ions released from intracellular metalloproteins. In contrast, probe **B** exhibits selectivity but only moderate sensitivity to Zn(II) ions, and our theoretical calculations did not provide an answer for this difference.

## Figures and Tables

**Table 1:** Optical properties of intermediates and fluorescent probes **A** and **B**.

	Solvent	λ_abs_ (nm)	λ_em_ (nm)	ε_max_ (104 M^−1^cm^−1^)	Φ_f_ (%)
**Compound 4**	Buffer (pH7.0)	687	703	3.2 × 10^4^	1.4
	Ethanol	705	718	5.5 × 10^4^	6.7
	THF	708	725	5.6 × 10^4^	7.0
**Probe A**	Buffer (pH7.0)	695	701	3.0 × 10^4^	0.6
	Ethanol	707	717	4.6 × 10^4^	6.0
	THF	710	723	3.1 × 10^4^	6.8
**Compound 9**	Buffer (pH7.0)	685	712	3.5 × 10^4^	1.6
	Ethanol	705	717	6.1 × 10^4^	5.2
	THF	706	721	4.2 × 10^4^	7.5
**Probe B**	Buffer (pH7.0)	688	709	4.1 × 10^4^	0.8
	Ethanol	705	714	6.0 × 10^4^	4.1
	THF	717	719	5.1 × 10^4^	5.0

## Supplementary Materials

The following are available online at https://www.mdpi.com/1420-3049/24/8/1592/s1, The supplementary materials include high-resolution mass spectra of probes **A** and **B** in the absence and presence of Zn(II) ions, Zn(II) binding constants with the probes, probe selectivity over other metal ions, Job’s plot, fluorescence quantum yields of the probes, and theoretical calculation results.
