# Supplementary material for: Detecting Zn(II) Ions in Live Cells with Near-Infrared Fluorescent Probes
Source: Molecules. 2019 Apr 22;24(8):1592. doi: 10.3390/molecules24081592 (PMC6515227; doi:10.3390/molecules24081592)
Supplement: Supplementary file 1 [file molecules-24-01592-s001.pdf]

Supplementary Materials

Detecting Zn(II) Ions in Live Cells with Near-Infrared Fluorescent probes

Mingxi Fang,[1] Shuai Xia,[1] Jianheng Bi,[1] Travis P. Wigstrom,[1] Loredana Valenzano,[1] Jianbo Wang,[1,2,*] Marina Tanasova,[1] Rudy L. Luck[1,*] and Haiying Liu[1*]

1 Department of Chemistry, Michigan Technological University, 1400 Townsend Drive, Houghton, MI 49931, USA; mfang@mtu.edu (M.F.); shuaix@mtu.edu (S.X.); jbi1@mtu.edu (J.B.); tpwigstr@mtu.edu (T.P.W.); lvalenza@mtu.edu (L.V.); mtanasov@mtu.edu (M.T.)

2 College of Biological, Chemical Sciences and Engineering, Jiaxing University, Jiaxing 314001, China

* Correspondence: wjb4207@mail.ustc.edu.cn (J.W.); rluck@mtu.edu (R.L.L.); hyliu@mtu.edu (H.L)

Received: 25 March 2019.; Accepted: 04 April 2019.; Published: date

Contents

1. HRMS spectra of fluorescent probes **A** and **B** 4

Figure S1. High resolution mass spectrum of compound **4**. 4
Figure S2. High resolution mass spectrum of probe **A**. 4
Figure S3. High resolution mass spectrum of compound **9**. 5
Figure S4. High resolution mass Spectrum of probe **B**. 5
Figure S5. High resolution mass spectrum of compound **8**. 6

2. ESI-MS of probes A binding with Zn(II) ions 7

Figure S6. ESI-MS of probe **A** binding with Zn^{2+} 7

3. Determination of binding constant of probe A 7

Figure S7. Benesi-Hildbrand equation plot of 5 μM fluorescent probe **A** with Zn(II) obtained from fluorescence titration data. The binding constant was determined to be 9.5×105 M-1. 8

4. Determination of detection limit of probe A 8

Figure S8. Fluorescence intensities of 10 μM fluorescent probe **A** at 701 nm as a function of the concentrations of Zn(II) in HEPES buffer solution (10 mM, pH 7.0). The detection limit was then determined to be 4.5×10-10 M. 9

5. Selectivity and photostability of probes A and B 10

Figure S9. (a) Fluorescence responses of probes **A** to Zn(II) ions over other metal ions, (b) Fluorescence responses of probe **B** to Zn(II) ions and other metal ions. 10
Figure S10. (a) Normalized photostability of probes **A** and (b) probe **B** with 2.0 μM Zn(II) as a function of time in 180 minutes under excitation (635 nm) in 10 mM HEPES buffer solutions (pH 7.0). 10

6. Job's plot 11

Figure S11. Job's plot of the complexation of fluorescent probe A with Zn(II) was plotted as function of the different molar ratio [A]/([A]+[Zn(II)]) by fluorescence method, indicating that there is 1:1 stoichiometry between Zn(II) and probe A. The total concentration of probe **A** and Zn(II) was constantly maintained in 2×10^{-5}M in the HEPES buffer solution (10mM, pH 7.0). 11

7. Fluorescence quantum yield 11

8. In-vitro cell imaging and intracellular detection of Zn(II) by using probe B 12

Figure S12. Fluorescence images of fluorescent probe **B** with concentration at 0.1 μM, 0.5 μM and 1.0 μM in HeLa cells. Cells were incubated with of probe **B** with specific concentration for 30 min. Cells were then supplemented with either 10 μM of zinc (II) chloride or 10 μM each of zinc (II) chloride plus sodium pyrithione (Pyr) for 30 min before acquiring images. Scale bar: 50 μm. λex: 635 nm. 12

9. Theoretical Calculation Results 12

Theoretical Data for probe **A + $Zn(OH_2)^{2+}$.** 12

Figure S13. GaussView representation of probe **A + $Zn(OH_2)^{2+}$**. 12

Figure S14. Illustration of the computational results for probe **A + $Zn(OH_2)^{2+}$.** 13

Table S1. Calculated atomic coordinates for probe **A + $Zn(OH_2)^{2+}$.** 13

Figure S15. Calculated IR spectrum for probe **A + $Zn(OH_2)^{2+}$.** 17

Figure S16. Calculated UV-Vis spectrum for probe **A + $Zn(OH_2)^{2+}$**. 17

Table S2. Excitation energies and oscillator strengths listing for probe **A + $Zn(OH_2)^{2+}$** 18

Theoretical Data for probe **A + $Zn(OH_2)_2^{2+}$.** 19

Figure S17. GaussView representation of probe **A + $Zn(OH_2)_2^{2+}$.** 19

Figure S18. Illustration of the computational results for probe **A + $Zn(OH_2)_2^{2+}$**. 19

Table S3. Calculated atomic coordinates for probe **A + $Zn(OH_2)_2^{2+}$**. 19

Figure S19. Calculated IR spectrum for probe **A + $Zn(OH_2)_2^{2+}$**. 23

Figure S20. Calculated UV-Vis spectrum for probe **A + $Zn(OH_2)_2^{2+}$**. 23

Table S4. Excitation energies and oscillator strengths listing for probe **A + $Zn(OH_2)_2^{2+}$**. 24

Theoretical Data for probe **B + $Zn(OH_2)^{2+}$**. 25

Figure S21. GaussView representation of probe **B + $Zn(OH_2)^{2+}$.** 25

Figure S22. Illustration of the computational results for probe **B + $Zn(OH_2)^{2+}$.** 25

Table S5. Calculated atomic coordinates for probe **B + $Zn(OH_2)^{2+}$**. 25

Figure S23. Calculated UV-Vis spectrum for probe **B + $Zn(OH_2)^{2+}$.** 29

Figure S24. Calculated UV-Vis spectrum for probe **B + $Zn(OH_2)^{2+}$.** 29

Table S6. Excitation energies and oscillator strengths listing for probe **B + $Zn(OH_2)^{2+}$**. 29

Theoretical Data for probe **B + $Zn(OH_2)_2^{2+}$.** 30

Figure S25. GaussView representation of probe **B + $Zn(OH_2)_2^{2+}$.** 30

Figure S26. Illustration of the computational results for probe **B + $Zn(OH_2)_2^{2+}$.** 31

Table S7. Computational results for probe **B + $Zn(OH_2)_2^{2+}$**. 31

Figure S27. Calculated IR spectrum for probe **B + $Zn(OH_2)_2^{2+}$**. 34

Figure S28. Calculated UV-Vis spectrum for probe **B + $Zn(OH_2)_2^{2+}$.** 34

Table S8. Excitation energies and oscillator strengths listing for probe **B + $Zn(OH_2)_2^{2+}$.** 35

Table S9. Calculated electronic transitions (nm) and corresponding oscillator strengths (f) and their percentage contribution to the UV/Vis spectra. 36

Table S10. Drawings of the HOMO-1/LUMO states for Intermediates **4** and **9**, and for probes **A** and **B**. 37

Table S11. Drawings of the HOMO-1/LUMO distributions for the Zn complexes explored in this work. Note that exceptions are observed for (b) and (f), corresponding to Zn complex **4**, for which the excitation is predicted as an HOMO/LUMO transition. 37

Table S12. Calculated electronic transitions (nm) and corresponding oscillator strengths (f) and their percentage contribution to the UV/Vis spectra for the $[Zn(OH_2)]^{2+}$ and $[Zn(OH_2)_2]^{2+}$ moieties attached to probes **A** and **B**. 38

Figure S29. Calculated coordination bond distances (Å) for probes **A** and **B** with $Zn(OH_2)^{2+}$ and $Zn(OH_2)_2^{2+}$. 38

10. Comparison of Zn concentration via atomic absorption spectroscopy and fluorescence change 39

Figure S30. Calibration curve of atomic absorption spectroscopy of Zn element. 39

Figure S31. Absorption of stock solution samples obtained in atomic absorption spectroscopy and calibration curve; (a) Zn^{2+} concentration of sample **1** was calculated to be 0.06mg/L= 0.923μM, (b) sample **2** was calculated to be 0.0438mg/L= 0.673μM. 40

Figure S32. Determination of Zn^{2+} concentration according to fluorescence changes. 40

11. References 41

1. HRMS spectra of fluorescent probes **A** and **B**

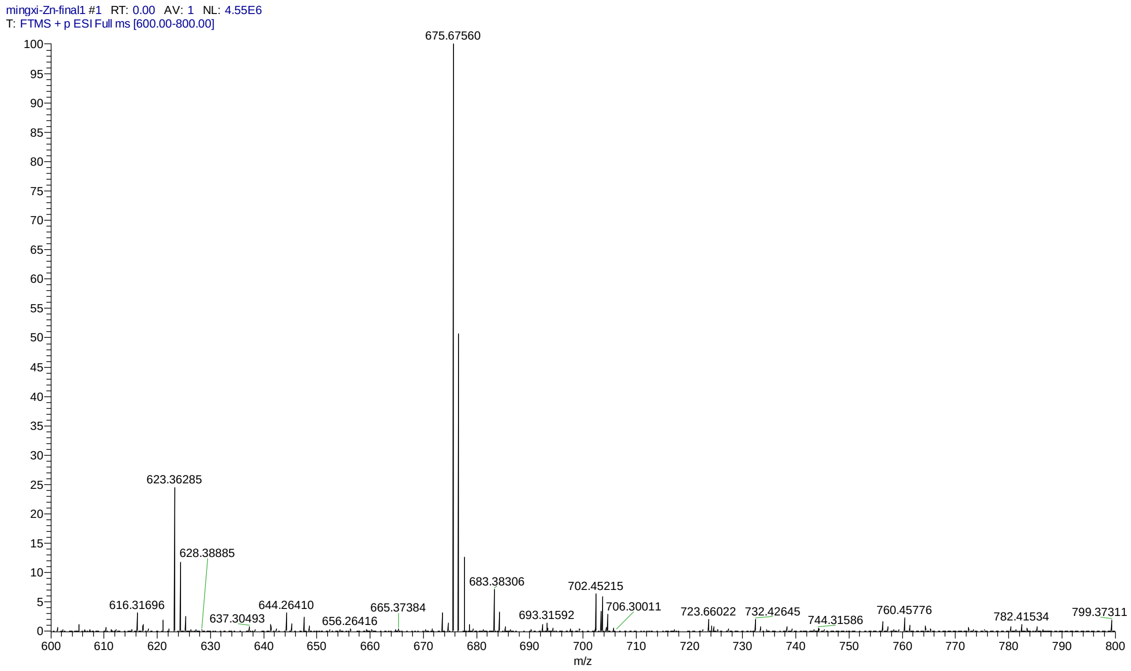

Figure S1. High resolution mass spectrum of compound **4**.

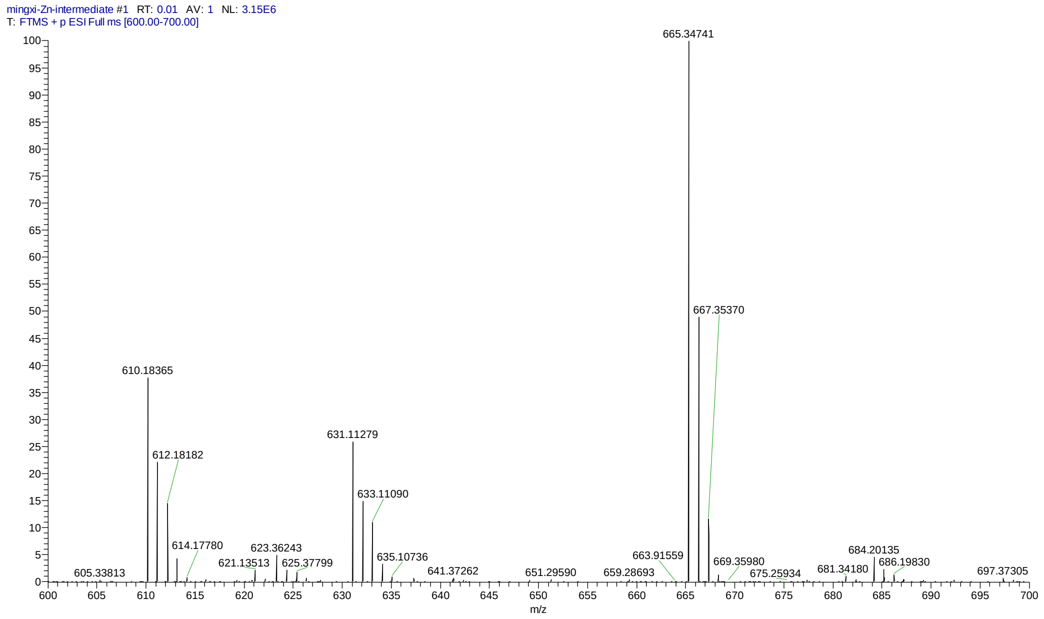

Figure S2. High resolution mass spectrum of probe **A**.

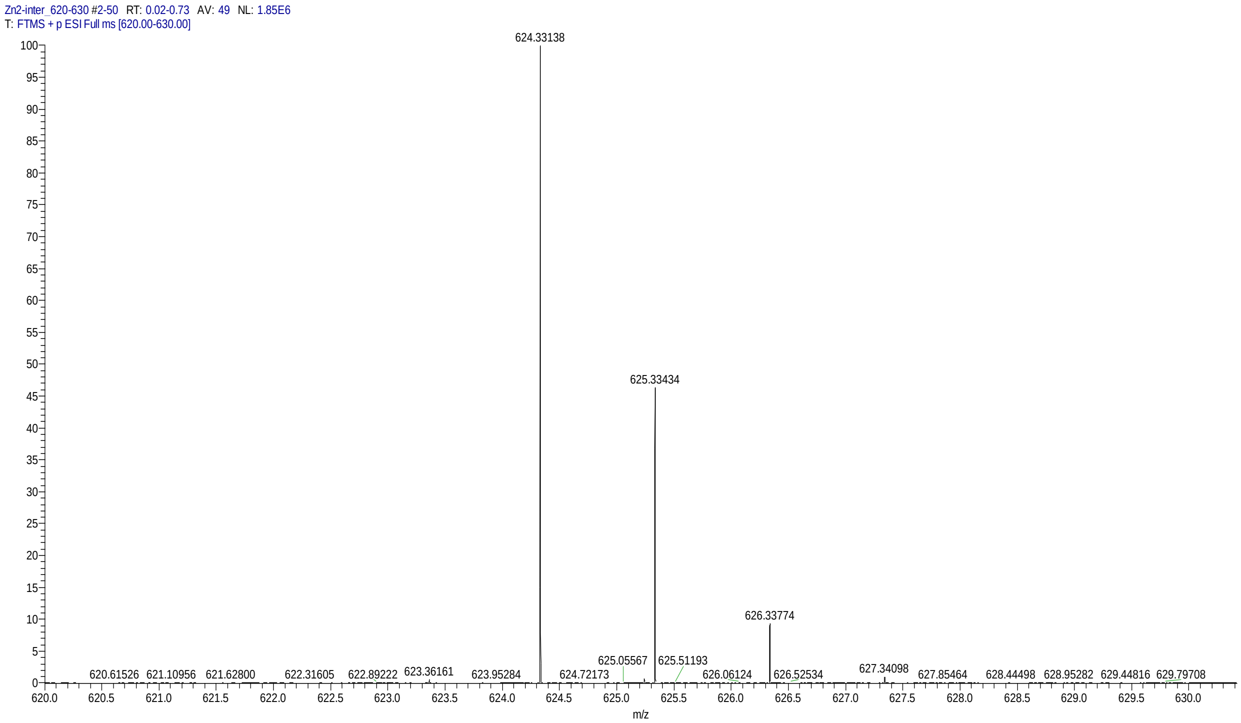

Figure S3. High resolution mass spectrum of compound **9**.

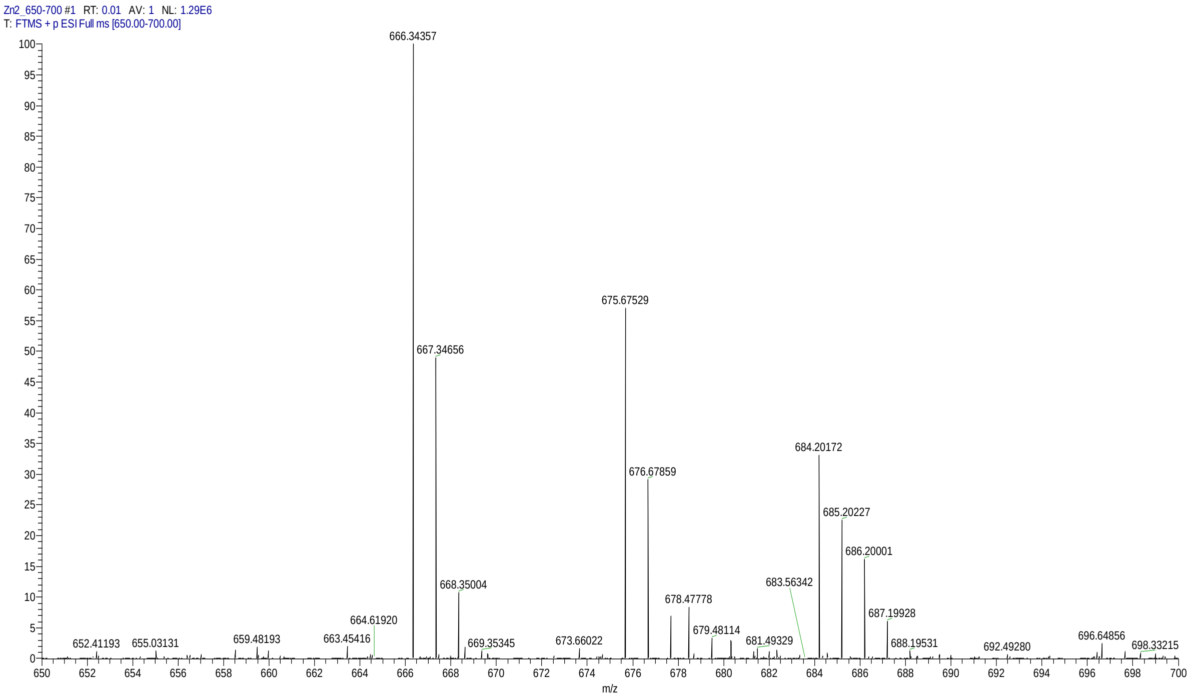

Figure S4. High resolution mass Spectrum of probe **B**.

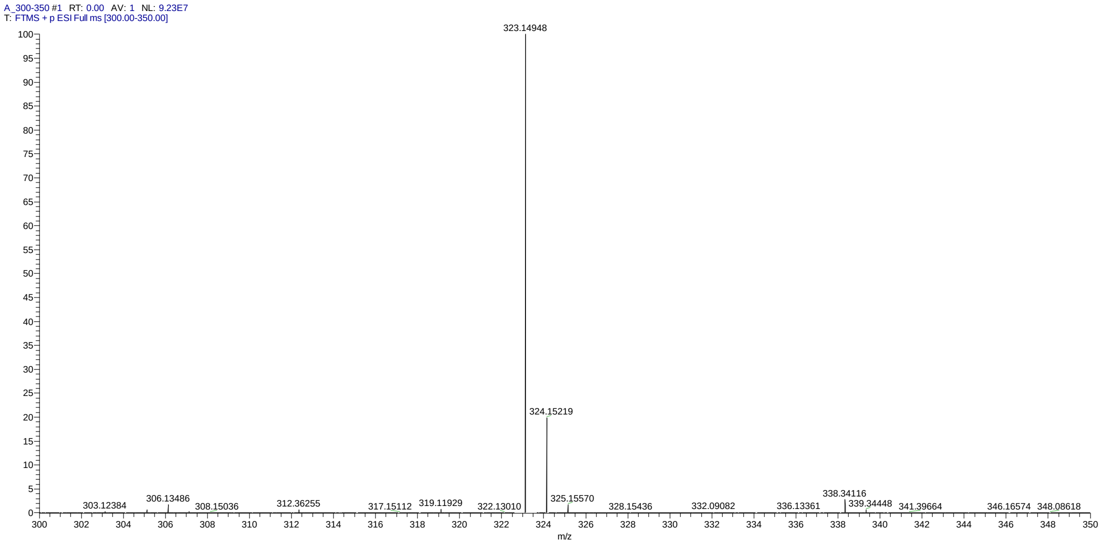

Figure S5. High resolution mass spectrum of compound **8**.

2. ESI-MS of probes A binding with Zn(II) ions

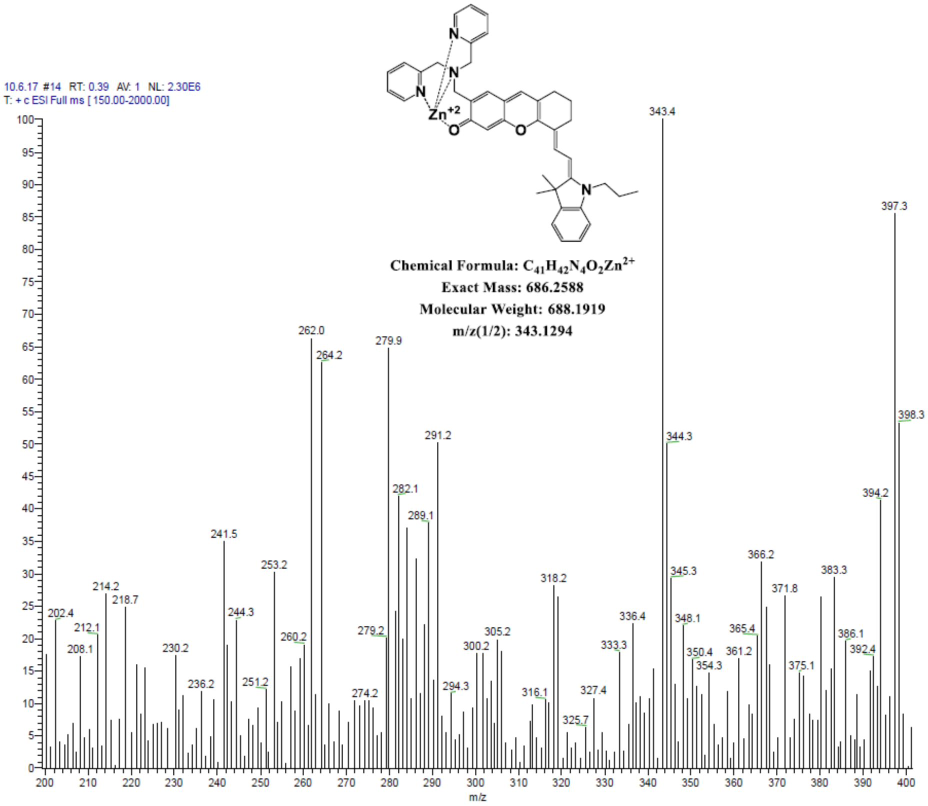

Figure S6. ESI-MS of probe **A** binding with Zn^{2+}

3. Determination of binding constant of probe A

Binding constant (K_a) was calculated by fluorescence method based on the modified Benesi-Hildbrand equation/plot. Fluorescent intensities were collected from the titration curves of the fluorescent probes **A** and **B** in the presence of Zn(II). K_a was determined from the slope of the fitting line and the intercept by using the following equation[1]:

$$\frac{1}{F - F_{min}} = \frac{1}{K_a[L](F_{max} - F_{min})} + \frac{1}{F_{max} - F_{min}}$$

Where F_{min}, F, and F_{max} are the fluorescence intensities of receptor considered in the absence of Zn(II), at an intermediate Zn(II) concentration, and at a concentration of complete saturation. K_a is the binding constant and $[L]$ is the Zn(II) concentration, respectively. K_a is determined from the slope and intercept of the linear regression equation. According to the plot of $\frac{1}{F-F_{min}}$ verses $\frac{1}{[L]}$, the good linear relationship (R=0.99) of fluorescent probes **A** and **B** also indicated the formation of 1:1 complexation.

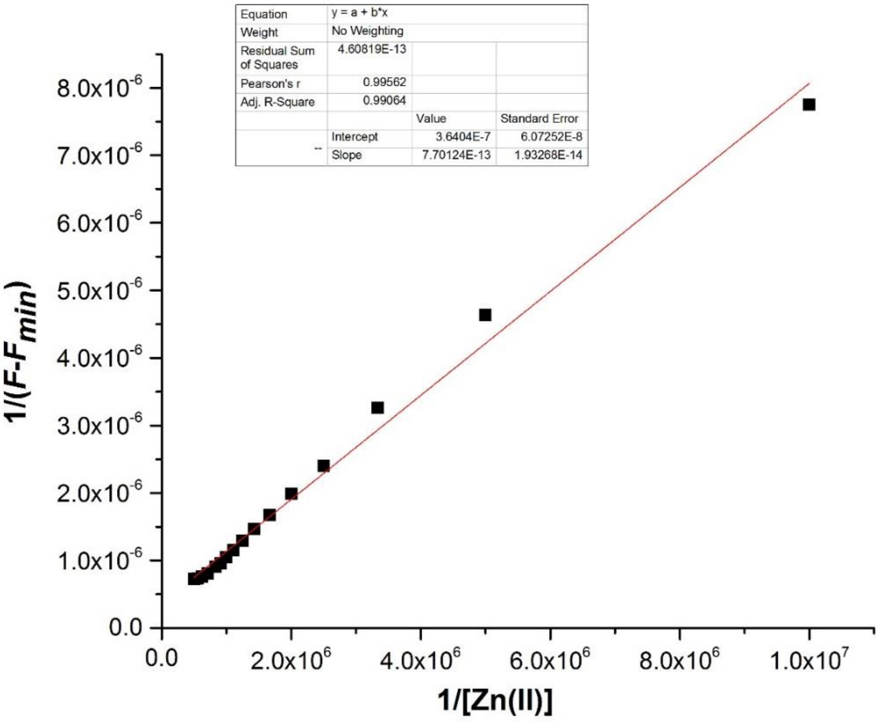

Figure S7. Benesi-Hildbrand equation plot of 5 μM fluorescent probe **A** with Zn(II) obtained from fluorescence titration data. The binding constant was determined to be 9.5×105 M-1.

4. Determination of detection limit of probe A

Detection limit (DL) of fluorescent probe **A** or **B** was calculated based on fluorescence titration by using the following formula[2]:

$$\mathrm{DL}=\mathrm{K}\times\frac{\sigma}{s}$$

Where K=3; s is the slope of the linear regression equation; δ is the standard deviation of the blank solution, which was measured 20 times to determine the background noise.

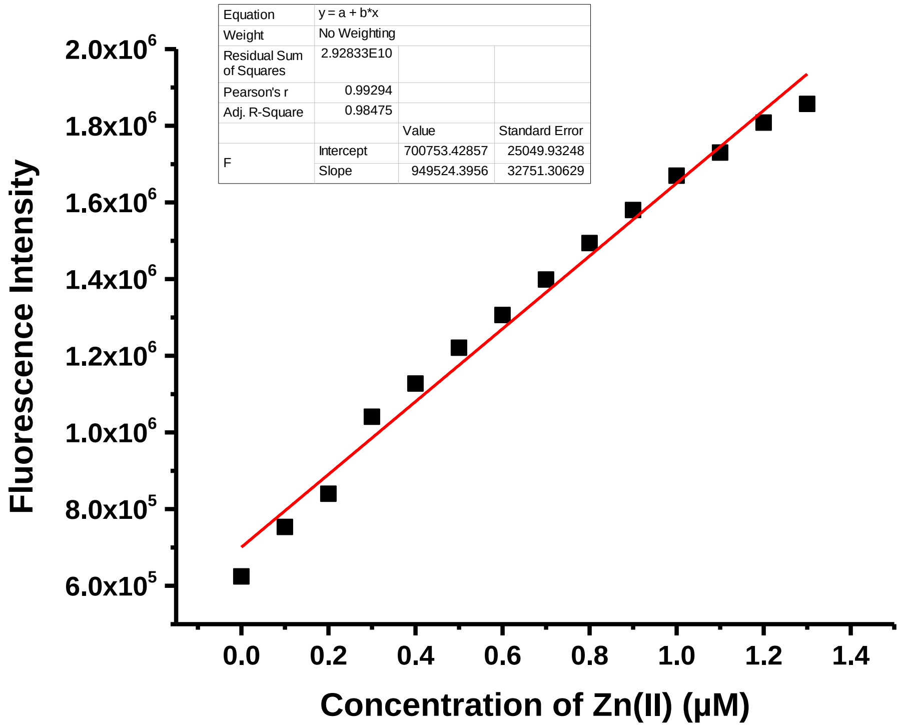

Figure S8. Fluorescence intensities of 10 μM fluorescent probe A at 701 nm as a function of the concentrations of Zn(II) in HEPES buffer solution (10 mM, pH 7.0). The detection limit was then determined to be 4.5×10-10 M.

5. Selectivity and photostability of probes A and B

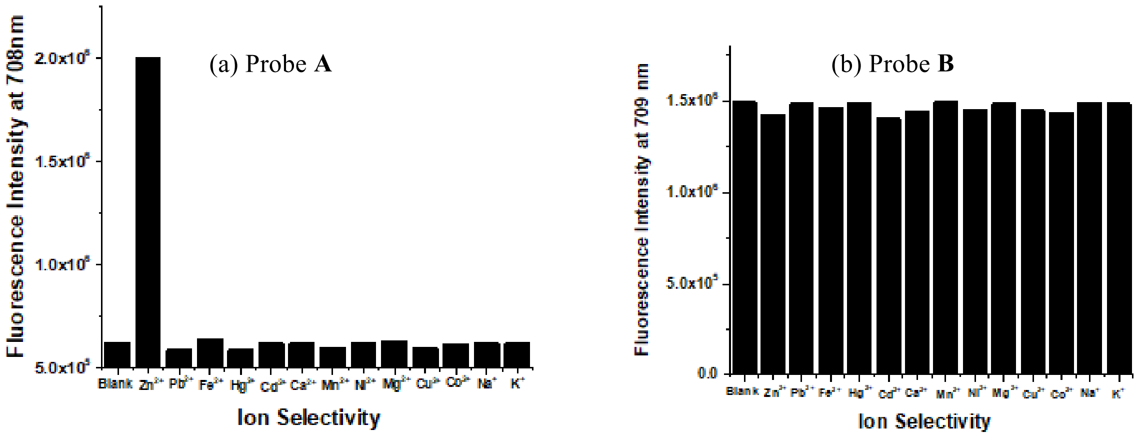

Figure S9. (a) Fluorescence responses of probes **A** to Zn(II) ions over other metal ions, (b) Fluorescence responses of probe **B** to Zn(II) ions and other metal ions.

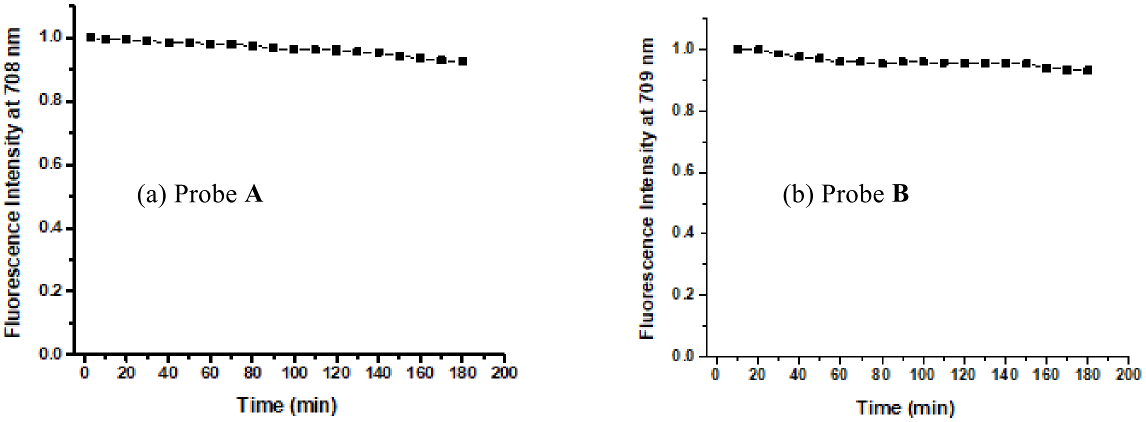

Figure S10. (a) Normalized photostability of probes **A** and (b) probe **B** with 2.0 μM Zn(II) as a function of time in 180 minutes under excitation (635 nm) in 10 mM HEPES buffer solutions (pH 7.0).

6. Job's plot

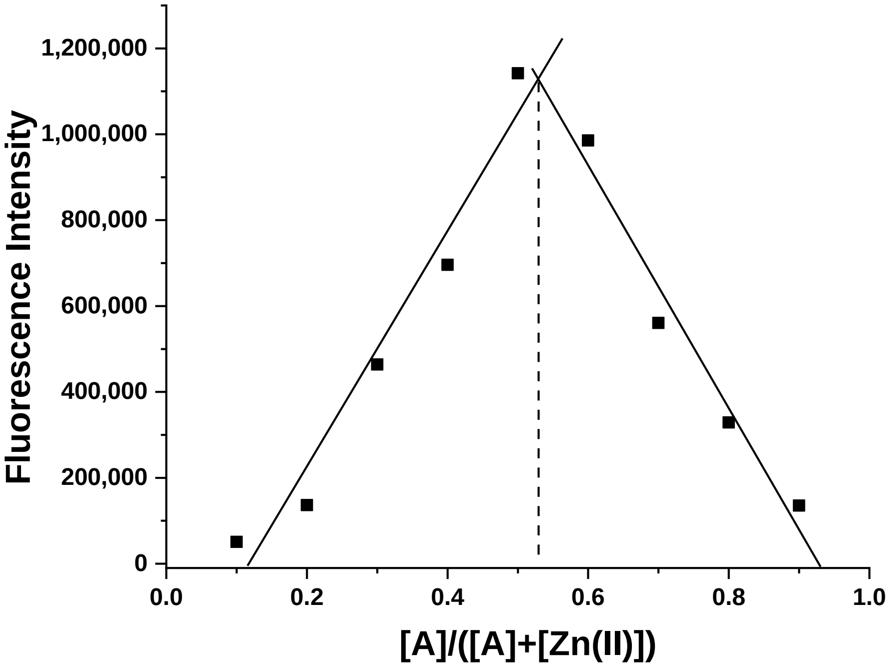

Figure S11. Job's plot of the complexation of fluorescent probe A with Zn(II) was plotted as function of the different molar ratio [A]/([A]+[Zn(II)]) by fluorescence method, indicating that there is 1:1 stoichiometry between Zn(II) and probe A. The total concentration of probe **A** and Zn(II) was constantly maintained in 2×10^{-5}M in the HEPES buffer solution (10mM, pH 7.0).

7. Fluorescence quantum yield

The quantum yields were calculated using Hunan Dye (QY = 0.41 in ethanol) as reference according to the method reported in literature[3].

$$QY = \frac{\mathbf{QY_r} \times \mathbf{I_x} \times \mathbf{A_r} \times \mathbf{n_x^2}}{\mathbf{I_r} \times \mathbf{A_x} \times \mathbf{n_r^2}}$$

QY_r: Quantum yield of reference compound.

A_x: Absorbance value of probe at excitation wavelength

I_x: Integration of probe's emission spectra

A_r: Absorbance value of the standard at excitation wavelength

I_r: Integration of standard emission spectra

n_s: Refraction rate of reference compound solvent.

n_x: Refractive index of probe solvent.

8. In-vitro cell imaging and intracellular detection of Zn(II) by using probe B

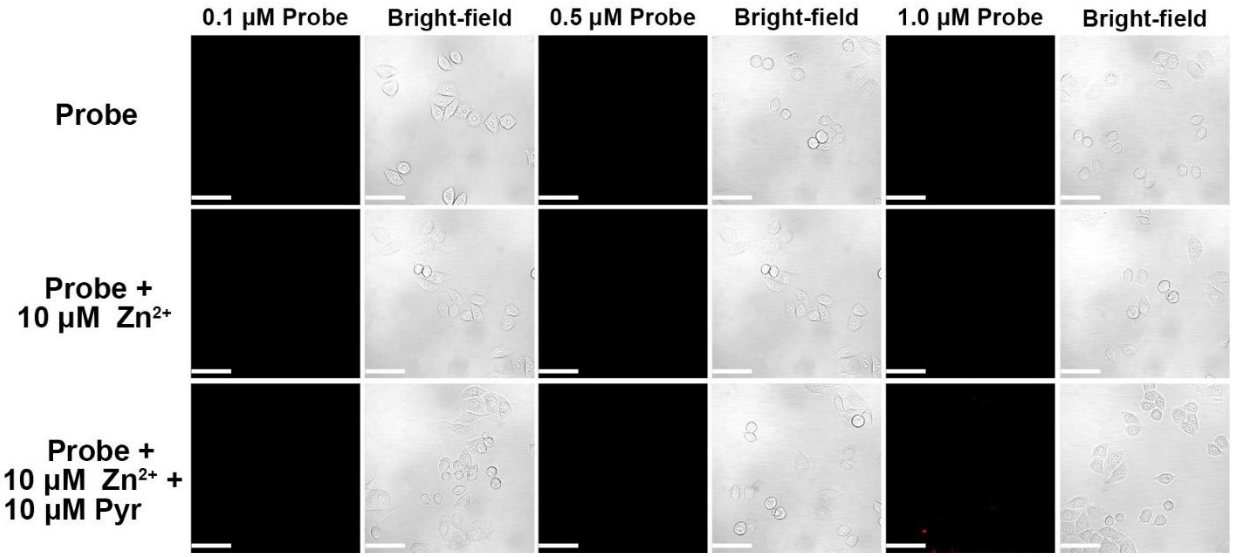

Figure S12. Fluorescence images of fluorescent probe **B** with concentration at 0.1 µM, 0.5 µM and 1.0 µM in HeLa cells. Cells were incubated with of probe **B** with specific concentration for 30 min. Cells were then supplemented with either 10 µM of zinc (II) chloride or 10 µM each of zinc (II) chloride plus sodium pyrithione (Pyr) for 30 min before acquiring images. Scale bar: 50 µm. λex: 635 nm.

9. Theoretical Calculation Results

Theoretical Data for probe **A + $Zn(OH_2)^{2+}$.**

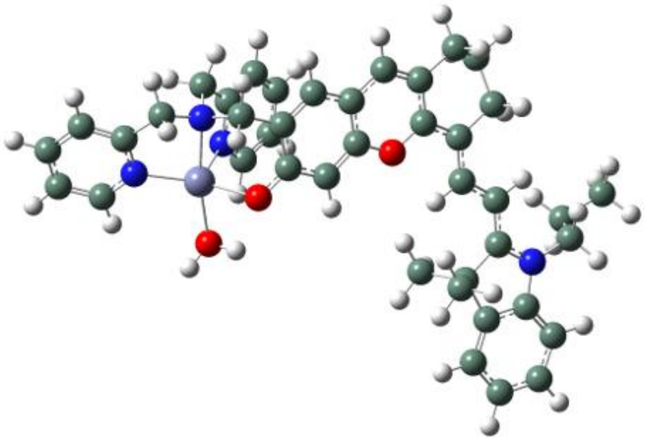

Figure S13. GaussView representation of probe **A + $Zn(OH_2)^{2+}$**.

zna1 (Optimization completed)		
/home/rluck/calculation/liu/mingxi/superior/zna1....		
File Type	.log	
Calculation Type	FREQ	
Calculation Method	RAPFD	
Basis Set	6-311+G(2d,p)	
Charge	2	
Spin	Singlet	
Solvation	scrf=solvent=water	
E(RAPFD)	-3811.478986	Hartree
RMS Gradient Norm	0.000004	Hartree/Bohr
Imaginary Freq		
Dipole Moment	17.364108	Debye
Point Group	C1	
Job cpu time:	21 days 14 hours 29 minutes 3...	

Figure S14. Illustration of the computational results for probe **A + $Zn(OH_2)^{2+}$.**

Table S1. Calculated atomic coordinates for probe **A + $Zn(OH_2)^{2+}$.**

Row	Symbol	X	Y	Z
1	C	−6.00124	−4.07936	−0.5792
2	C	−7.22448	−4.73893	−0.44846
3	C	−8.38702	−4.02386	−0.17466
4	C	−8.36582	−2.63846	−0.02846
5	C	−7.1402	−2.00844	−0.17024
6	C	−5.97027	−2.70785	−0.43404
7	N	−6.84182	−0.64385	−0.08277
8	C	−5.52456	−0.41429	−0.23769
9	C	−4.81919	−1.73859	−0.51019
10	C	−7.84449	0.362558	0.212029
11	C	−7.99077	0.610224	1.708249
12	C	−9.03492	1.680775	1.988609
13	C	−3.78453	−2.06766	0.575385
14	C	−4.19829	−1.77004	−1.91477
15	C	−4.97094	0.853266	−0.14087
16	C	−3.62283	1.132249	−0.3309
17	C	−3.04128	2.39066	−0.2589
18	C	−3.84174	3.626272	0.066536
19	C	−2.96914	4.771134	0.568196
20	C	−1.82188	5.030059	−0.39939
21	C	−1.01236	3.784132	−0.60906
22	C	−1.67185	2.533534	−0.52803
23	C	0.325985	3.8008	−0.89321

Row	Symbol	X	Y	Z
24	C	1.048795	2.608601	−1.10755
25	C	0.348218	1.392947	−1.01372
26	O	−0.97704	1.395492	−0.72774
27	C	2.42451	2.541164	−1.38041
28	C	3.06697	1.337964	−1.52832
29	C	2.33507	0.108375	−1.42033
30	C	0.9548	0.169089	−1.176
31	O	2.942398	−1.03901	−1.51336
32	C	4.541643	1.267092	−1.7664
33	N	5.258928	0.651113	−0.6285
34	C	7.222279	−0.75681	−0.17334
35	C	3.843877	1.06278	1.350535
36	C	3.085038	2.010567	2.018689
37	C	1.949641	1.603011	2.700043
38	C	1.606882	0.257293	2.703694
39	C	2.412917	−0.62742	2.012529
40	N	3.505659	−0.23139	1.35385
41	C	8.568658	−0.83158	0.146162
42	C	8.164438	−2.97732	1.125465
43	C	6.833459	−2.82709	0.787053
44	N	6.376181	−1.74391	0.149536
45	Zn	4.396447	−1.3257	−0.17359
46	C	6.662724	0.405728	−0.95088
47	C	5.107272	1.419979	0.61378
48	H	−5.09493	−4.63866	−0.78858
49	H	−7.26953	−5.81707	−0.55732
50	H	−9.3292	−4.55119	−0.06969
51	H	−9.27518	−2.09236	0.193121
52	H	−7.57663	1.279876	−0.31532
53	H	−8.78777	0.024049	−0.22063
54	H	−8.26364	−0.32954	2.199566
55	H	−7.01919	0.906787	2.116992
56	H	−9.1425	1.852892	3.061703
57	H	−8.75931	2.631876	1.523765
58	H	−10.0143	1.390388	1.597375
59	H	−3.40239	−3.0781	0.41442
60	H	−2.93962	−1.37887	0.55436
61	H	−4.23744	−2.02688	1.567967
62	H	−4.94268	−1.52222	−2.67401
63	H	−3.36672	−1.07095	−2.00742
64	H	−3.82191	−2.77463	−2.1192
65	H	−4.3999	3.948142	−0.82268
66	H	−4.59186	3.379549	0.82364
67	H	−2.22694	5.362391	−1.36401
68	H	−1.17048	5.831033	−0.04054
69	H	2.986074	3.46858	−1.4491

Row	Symbol	X	Y	Z
70	H	4.945727	2.266633	−1.96776
71	H	4.743695	0.636957	−2.63616
72	H	0.725054	−0.10174	3.219776
73	H	2.185521	−1.6859	1.962097
74	H	6.10174	−3.58732	1.033348
75	H	7.282683	1.296019	−0.80046
76	H	6.724395	0.154323	−2.01401
77	H	5.952517	1.179217	1.266309
78	H	5.146928	2.497238	0.420834
79	O	3.248971	−3.12672	0.163435
80	H	0.383732	−0.74632	−1.07386
81	H	0.849631	4.750739	−0.957
82	H	−3.57172	5.674017	0.694431
83	H	−2.56095	4.515045	1.552522
84	H	−5.63708	1.674742	0.093198
85	H	−2.96212	0.311501	−0.57498
86	H	3.618502	−4.00319	0.005638
87	H	2.616841	−2.96301	−0.55386
88	C	9.047015	−1.95643	0.799293
89	H	8.494322	−3.87078	1.640614
90	H	10.09728	−2.03285	1.058085
91	H	9.228289	−0.01282	−0.11655
92	H	3.373436	3.054522	1.98364
93	H	1.332425	2.329971	3.216607
59	H	−3.40239	−3.0781	0.41442
60	H	−2.93962	−1.37887	0.55436
61	H	−4.23744	−2.02688	1.567967
62	H	−4.94268	−1.52222	−2.67401
63	H	−3.36672	−1.07095	−2.00742
64	H	−3.82191	−2.77463	−2.1192
65	H	−4.3999	3.948142	−0.82268
66	H	−4.59186	3.379549	0.82364
67	H	−2.22694	5.362391	−1.36401
68	H	−1.17048	5.831033	−0.04054
69	H	2.986074	3.46858	−1.4491
70	H	4.945727	2.266633	−1.96776
71	H	4.743695	0.636957	−2.63616
72	H	0.725054	−0.10174	3.219776
73	H	2.185521	−1.6859	1.962097
74	H	6.10174	−3.58732	1.033348
75	H	7.282683	1.296019	−0.80046
76	H	6.724395	0.154323	−2.01401
77	H	5.952517	1.179217	1.266309
78	H	5.146928	2.497238	0.420834

Row	Symbol	X	Y	Z
79	O	3.248971	−3.12672	0.163435
80	H	0.383732	−0.74632	−1.07386
81	H	0.849631	4.750739	−0.957
82	H	−3.57172	5.674017	0.694431
83	H	−2.56095	4.515045	1.552522
84	H	−5.63708	1.674742	0.093198
85	H	−2.96212	0.311501	−0.57498
86	H	3.618502	−4.00319	0.005638
87	H	2.616841	−2.96301	−0.55386
88	C	9.047015	−1.95643	0.799293
89	H	8.494322	−3.87078	1.640614
90	H	10.09728	−2.03285	1.058085
91	H	9.228289	−0.01282	−0.11655
92	H	3.373436	3.054522	1.98364
93	H	1.332425	2.329971	3.216607

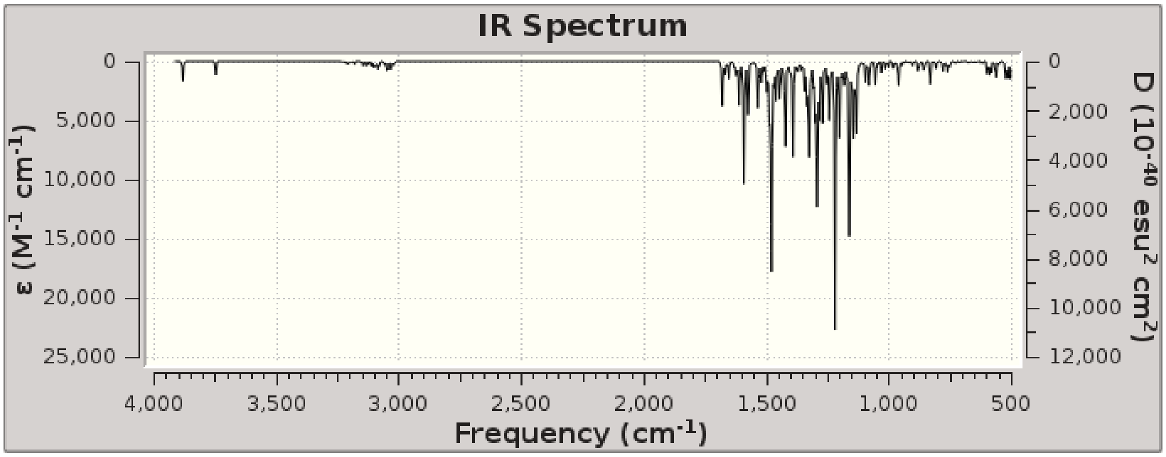

Figure S15. Calculated IR spectrum for probe **A + Zn(OH$_2$)$^{2+}$.**

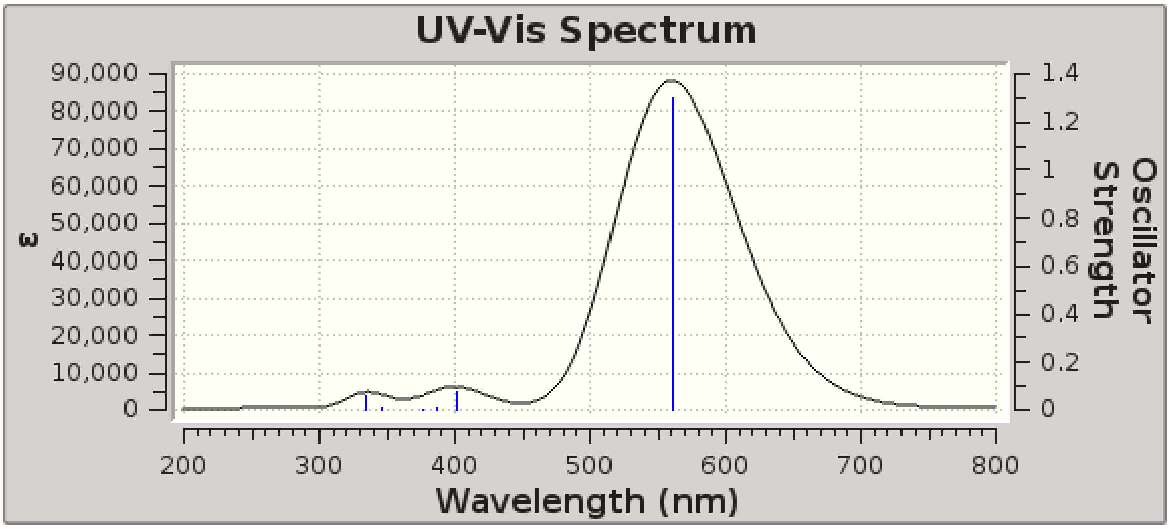

Figure S16. Calculated UV-Vis spectrum for probe **A + Zn(OH$_2$)$^{2+}$**.

Table S2. Excitation energies and oscillator strengths listing for probe **A + Zn(OH$_2$)$^{2+}$**.

Excited State	Nature	E (eV)	λ (nm)	*f*	Orbital transitions	Normalized coefficient
1	Singlet-A	2.2105	560.88	1.3017	185 → 186	0.70536
2	Singlet-A	3.0874	401.59	0.0791	183 → 186<br>184 → 186<br>185 → 188	0.14369<br>0.67231<br>0.10181
3	Singlet-A	3.2102	386.22	0.0073	185 → 187<br>185 → 188	−0.46017<br>0.52061
4	Singlet-A	3.2907	376.77	0.0013	185 → 187<br>185 → 188	0.52708<br>0.45346
5	Singlet-A	3.5768	346.63	0.0104	183 → 186<br>184 → 186<br>185 → 189<br>185 → 190	0.58152<br>−0.10464<br>0.33914<br>−0.10298
6	Singlet-A	3.7041	334.72	0.0562	183 → 186<br>185 → 189<br>185 → 190<br>185 → 191	−0.28429<br>0.49263<br>0.25723<br>−0.30559

Theoretical Data for probe **A + Zn(OH$_2$)$_2^{2+}$**

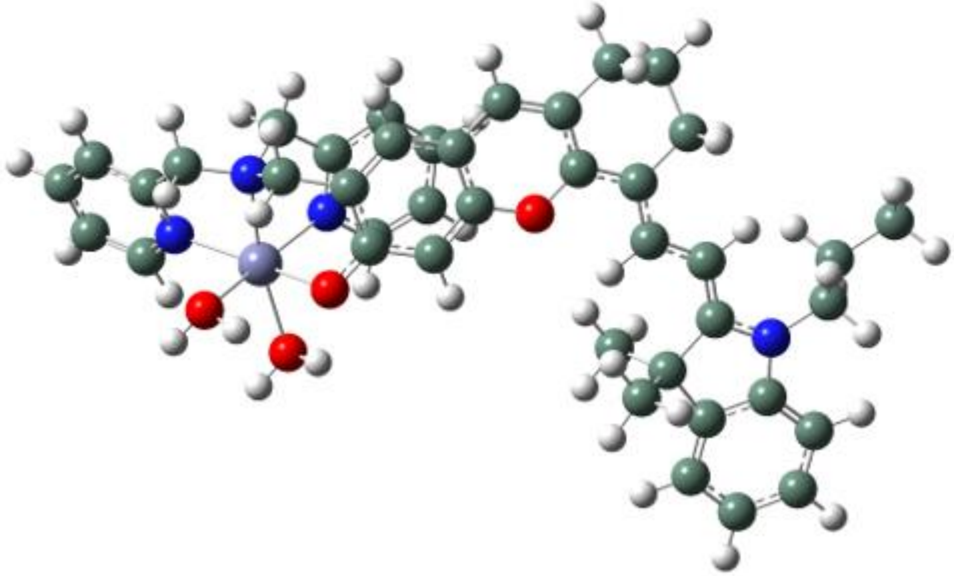

Figure S17. GaussView representation of probe **A + Zn(OH$_2$)$_2^{2+}$.**

zna3 (Optimization completed)		
/home/rluck/calculation/liu/mingxi/superior/zna3....		
File Type	.log	
Calculation Type	FREQ	
Calculation Method	RAPFD	
Basis Set	6-311+G(2d,p)	
Charge	2	
Spin	Singlet	
Solvation	scrf=solvent=water	
E(RAPFD)	-3887.894409	Hartree
RMS Gradient Norm	0.000006	Hartree/Bohr
Imaginary Freq		
Dipole Moment	15.969840	Debye
Point Group	C1	
Job cpu time: 24 days 8 hours 28 minutes 6...		

Figure S18. Illustration of the computational results for probe **A + Zn(OH$_2$)$_2^{2+}$**.

Table S3. Calculated atomic coordinates for probe **A + Zn(OH$_2$)$_2^{2+}$**.

Row	Symbol	X	Y	Z
1	C	-5.83834	-4.07314	-0.58017
2	C	-7.02655	-4.78335	-0.40013
3	C	-8.20427	-4.11817	-0.07122

Row	Symbol	X	Y	Z
4	C	-8.23351	-2.73371	0.082142
5	C	-7.0424	-2.05285	-0.10974
6	C	-5.85719	-2.70211	-0.42825
7	N	-6.79774	-0.67771	-0.02741
8	C	-5.49849	-0.39254	-0.23751
9	C	-4.75068	-1.68572	-0.54589
10	C	-7.82616	0.283528	0.323531
11	C	-7.91406	0.510745	1.827704
12	C	-8.98735	1.534208	2.167639
13	C	-3.66216	-1.97559	0.497966
14	C	-4.18347	-1.68806	-1.97317
15	C	-4.99312	0.894825	-0.14943
16	C	-3.66396	1.228029	-0.38496
17	C	3.11365	2.496398	-0.27082
18	C	-3.92431	3.690608	0.164528
19	C	-3.06116	4.784754	0.782922
20	C	-1.91416	5.151361	-0.14975
21	C	-1.1005	3.939212	-0.49671
22	C	-1.7535	2.685515	-0.56278
23	C	0.245497	3.988483	-0.74259
24	C	0.977894	2.829719	-1.06948
25	C	0.274495	1.614874	-1.16288
26	O	-1.05859	1.586242	-0.91803
27	C	2.370091	2.783728	-1.24817
28	C	3.021541	1.600462	-1.48663
29	C	2.281076	0.375517	-1.61864
30	C	0.885209	0.420787	-1.46325
31	O	2.895949	-0.74794	-1.81115
32	C	4.515455	1.531236	-1.4939
33	N	5.015449	0.78152	-0.31829
34	C	6.936033	-0.47967	0.527077
35	C	3.197388	0.786662	1.355536
36	C	2.219124	1.571742	1.947841
37	C	1.020377	0.98533	2.318613
38	C	0.834604	-0.37296	2.097531
39	C	1.856844	-1.08674	1.499803
40	N	3.010683	-0.51975	1.137774
41	C	8.167789	-0.4555	1.162419
42	C	8.561375	-1.55351	1.911479
43	C	7.708012	-2.6436	2.015506
44	C	6.490779	-2.58912	1.36195
45	N	6.117474	-1.53389	0.632438

Row	Symbol	X	Y	Z
46	Zn	4.299577	-1.26826	-0.36705
47	O	5.443859	-1.62484	-2.25423
48	C	6.472281	0.661312	-0.34067
49	C	4.536961	1.345799	0.953225
50	H	-4.91995	-4.5939	-0.832
51	H	-7.03189	-5.86196	-0.51337
52	H	-9.1184	-4.68446	0.072013
53	H	-9.15357	-2.22622	0.347092
54	H	-7.62026	1.21613	-0.20482
55	H	-8.77365	-0.08949	-0.06997
56	H	-8.12516	-0.4442	2.320185
57	H	-6.93786	0.843568	2.195478
58	H	-9.05186	1.691989	3.246392
59	H	-8.77312	2.500146	1.70108
60	H	-9.97108	1.206885	1.818879
61	H	-3.24308	-2.96749	0.315288
62	H	-2.84935	-1.25037	0.450784
63	H	-4.07841	-1.96039	1.50728
64	H	-4.96601	-1.46902	-2.7023
65	H	-3.3849	-0.95583	-2.0956
66	H	-3.77442	-2.6759	-2.19598
67	H	-5.67995	1.68516	0.127846
68	H	-2.98877	0.43937	-0.68779
69	H	-4.47507	4.095739	-0.69467
70	H	-4.67964	3.370465	0.887453
71	H	-3.67078	5.665528	0.999813
72	H	-2.65312	4.431595	1.73681
73	H	-2.31997	5.5877	-1.07163
74	H	-1.26542	5.909481	0.296367
75	H	0.769315	4.937923	-0.67261
76	H	2.937365	3.703971	-1.14027
77	H	0.308371	-0.49535	-1.5107
78	H	4.943344	2.54093	-1.50147
79	H	4.875937	1.000905	-2.37833
80	H	2.392239	2.631796	2.09116
81	H	0.23389	1.584168	2.765007
82	H	-0.08977	-0.86969	2.366031
83	H	1.758581	-2.14398	1.281353
84	H	8.803529	0.417423	1.069969
85	H	9.521112	-1.55519	2.416438
86	H	7.972843	-3.51826	2.596585
87	H	5.783385	-3.40881	1.41864

Row	Symbol	X	Y	Z
88	H	5.923497	-2.44676	-2.4061
89	H	4.751236	-1.57997	-2.92577
90	H	6.965357	1.59209	-0.03834
91	H	6.772581	0.450734	-1.37099
92	H	5.25767	1.079469	1.732452
93	H	4.499314	2.438902	0.911592
94	O	3.244548	-3.12034	-0.57735
95	H	2.662353	-2.82245	-1.29402
96	H	3.705809	-3.9017	-0.90331

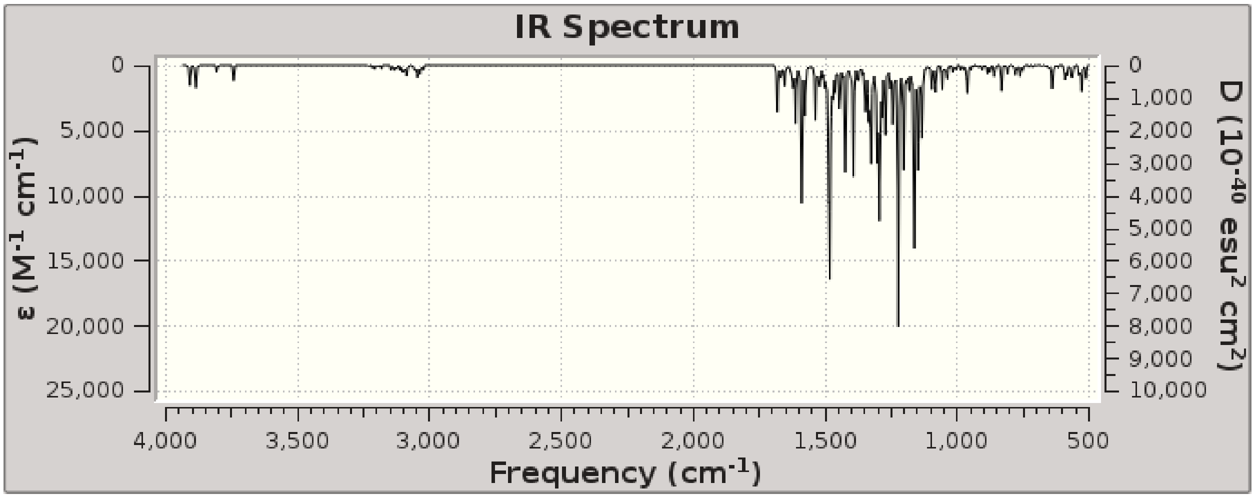

Figure S19. Calculated IR spectrum for probe **A + Zn(OH$_2$)$_2^{2+}$**.

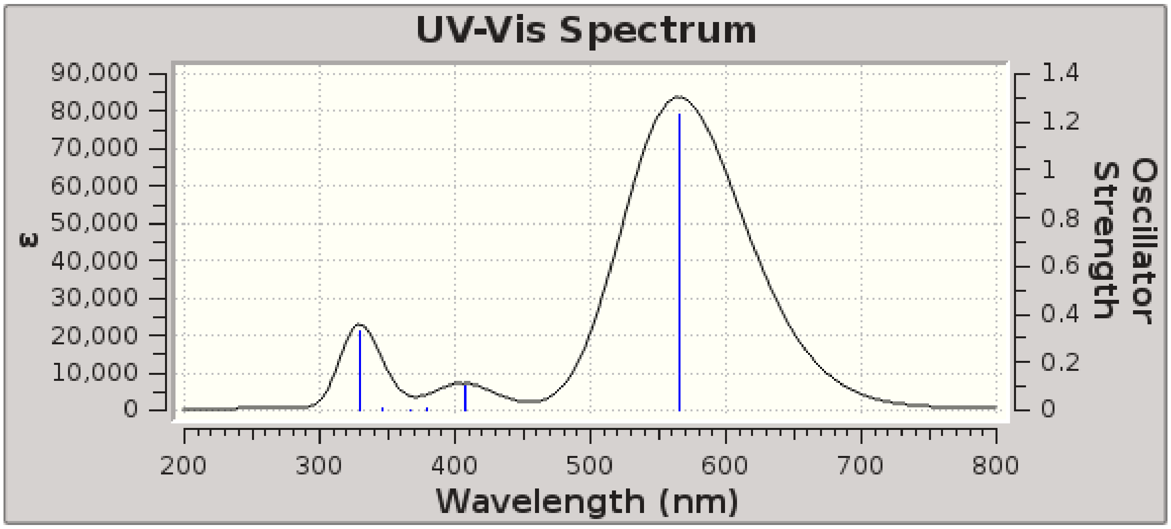

Figure S20. Calculated UV-Vis spectrum for probe **A + Zn(OH$_2$)$_2^{2+}$**.

Table S4. Excitation energies and oscillator strengths listing for probe **A + $Zn(OH_2)_2^{2+}$**.

Excited State	Nature	E (eV)	λ (nm)	*f*	Orbital transitions	Normalized coefficient
1	Singlet-A	2.1904	566.03	1.2351	190 → 191	0.70481
2	Singlet-A	3.0466	406.95	0.0977	188 → 191<br>189 → 191	0.13523<br>0.67983
3	Singlet-A	3.2716	378.98	0.0048	190 → 192<br>190 → 193	-0.22201<br>0.66178
4	Singlet-A	3.3757	367.29	0.0023	190 → 192<br>190 → 193	0.66241<br>0.21706
5	Singlet-A	3.5757	346.74	0.0067	188 → 191<br>190 → 194	0.60338<br>-0.31082
6	Singlet-A	3.7560	330.10	0.3288	188 → 191<br>189 → 191<br>190 → 194<br>190 → 195<br>190 → 196	0.31110<br>-0.11444<br>0.56920<br>-0.15345<br>-0.12199

Theoretical Data for probe **B + Zn(OH₂)²⁺**.

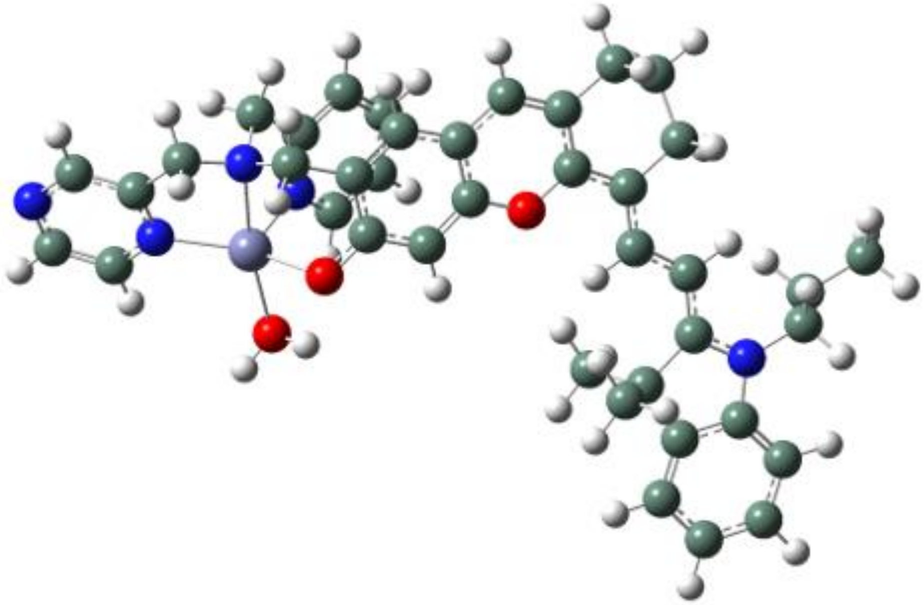

Figure S21. GaussView representation of probe **B + $Zn(OH_2)^{2+}$**.

znb1 (Optimization completed)		
/home/rluck/calculation/liu/mingxi/superior/crash...		
File Type	.log	
Calculation Type	FREQ	
Calculation Method	RAPFD	
Basis Set	6-311+G(2d,p)	
Charge	2	
Spin	Singlet	
Solvation	scrf=solvent=water	
E(RAPFD)	-3827.499827	Hartree
RMS Gradient Norm	0.000007	Hartree/Bohr
Imaginary Freq		
Dipole Moment	13.508812	Debye
Point Group	C1	
Job cpu time: 20 days 3 hours 42 minutes 2...		

Figure S22. Illustration of the computational results for probe **B + $Zn(OH_2)^{2+}$**.

Table S5. Calculated atomic coordinates for probe **B + $Zn(OH_2)^{2+}$**.

Row	Symbol	X	Y	Z
1	C	-5.99445	-4.0773	-0.57252
2	C	-7.2171	-4.73779	-0.44135
3	C	-8.38073	-4.02333	-0.17051
4	C	-8.36121	-2.63758	-0.02775

Row	Symbol	X	Y	Z
5	C	-7.13609	-2.0067	-0.16983
6	C	-5.96516	-2.70538	-0.43073
7	N	-6.83917	-0.64145	-0.08531
8	C	-5.52233	-0.41094	-0.23933
9	C	-4.81516	-1.73502	-0.50813
10	C	-7.84355	0.364295	0.206317
11	C	-7.99277	0.613389	1.701986
12	C	-9.03977	1.681927	1.979319
13	C	-3.78106	-2.0605	0.579093
14	C	-4.19315	-1.76899	-1.91219
15	C	-4.96979	0.857664	-0.14481
16	C	-3.62188	1.136105	-0.334
17	C	-3.03931	2.394616	-0.26439
18	C	-3.83876	3.631797	0.057192
19	C	-2.96528	4.776587	0.557573
20	C	-1.81598	5.031784	-0.40869
21	C	-1.00759	3.784444	-0.61416
22	C	-1.66977	2.534811	-0.53182
23	C	0.330972	3.798278	-0.89577
24	C	1.052221	2.603884	-1.1071
25	C	0.348904	1.390053	-1.01261
26	O	-0.9765	1.395265	-0.72844
27	C	2.427846	2.533277	-1.37869
28	C	3.067239	1.327861	-1.52519
29	C	2.332282	0.101116	-1.41707
30	C	0.952648	0.164416	-1.17315
31	O	2.938226	-1.0479	-1.51018
32	C	4.541123	1.250894	-1.76433
33	N	5.258721	0.636234	-0.62355
34	C	7.218096	-0.76605	-0.17564
35	C	3.843877	1.056773	1.354188
36	C	3.086876	2.006912	2.020729
37	C	1.952595	1.601834	2.705387
38	C	1.609189	0.256309	2.713892
39	C	2.413074	-0.63108	2.023958
40	N	3.504939	-0.23716	1.362013
41	C	8.568497	-0.86078	0.14372
42	C	8.235997	-2.88808	1.095852
43	C	6.884067	-2.81121	0.804747
44	N	6.386553	-1.7516	0.167668
45	Zn	4.380354	-1.33905	-0.1622
46	C	6.662795	0.397526	-0.94824

Row	Symbol	X	Y	Z
47	C	5.106846	1.411359	0.616045
48	H	-5.08735	-4.63615	-0.77962
49	H	-7.26088	-5.81624	-0.54754
50	H	-9.32242	-4.55147	-0.06518
51	H	-9.27137	-2.09189	0.191506
52	H	-7.57574	1.281231	-0.32165
53	H	-8.78557	0.024079	-0.22772
54	H	-8.26437	-0.32638	2.193981
55	H	-7.02249	0.912578	2.111908
56	H	-9.14928	1.855243	3.05202
57	H	-8.7657	2.63302	1.513569
58	H	-10.0179	1.388704	1.587074
59	H	-3.39803	-3.07103	0.420879
60	H	-2.93666	-1.37115	0.557031
61	H	-4.2348	-2.01765	1.571208
62	H	-4.93726	-1.52381	-2.67255
63	H	-3.36234	-1.06916	-2.00585
64	H	-3.8155	-2.77363	-2.11393
65	H	-4.39528	3.952357	-0.8335
66	H	-4.59022	3.387612	0.81379
67	H	-2.21901	5.362062	-1.37485
68	H	-1.16428	5.832912	-0.05077
69	H	2.991561	3.459275	-1.44836
70	H	4.949563	2.247828	-1.9685
71	H	4.740243	0.61655	-2.6316
72	H	0.728208	-0.10067	3.232769
73	H	2.184925	-1.68949	1.976951
74	H	7.282901	1.287036	-0.79392
75	H	6.727839	0.149396	-2.01198
76	H	5.95196	1.175869	1.270576
77	H	5.145598	2.487307	0.416915
78	O	3.222746	-3.12676	0.161587
79	H	0.379258	-0.74952	-1.07098
80	H	0.8565	4.747095	-0.9604
81	H	-3.56673	5.680639	0.680691
82	H	-2.55912	4.522205	1.543174
83	H	-5.63661	1.679295	0.086762
84	H	-2.9612	0.314559	-0.57535
85	H	3.574662	-4.00958	-0.00114
86	H	2.593098	-2.9466	-0.55454
87	N	9.078246	-1.9123	0.769853
88	H	3.37593	3.05052	1.982236

Row	Symbol	X	Y	Z
89	H	1.336735	2.330638	3.220908
90	H	9.247335	-0.05506	-0.11769
91	H	6.196861	-3.59821	1.088378
92	H	8.639865	-3.75285	1.610515

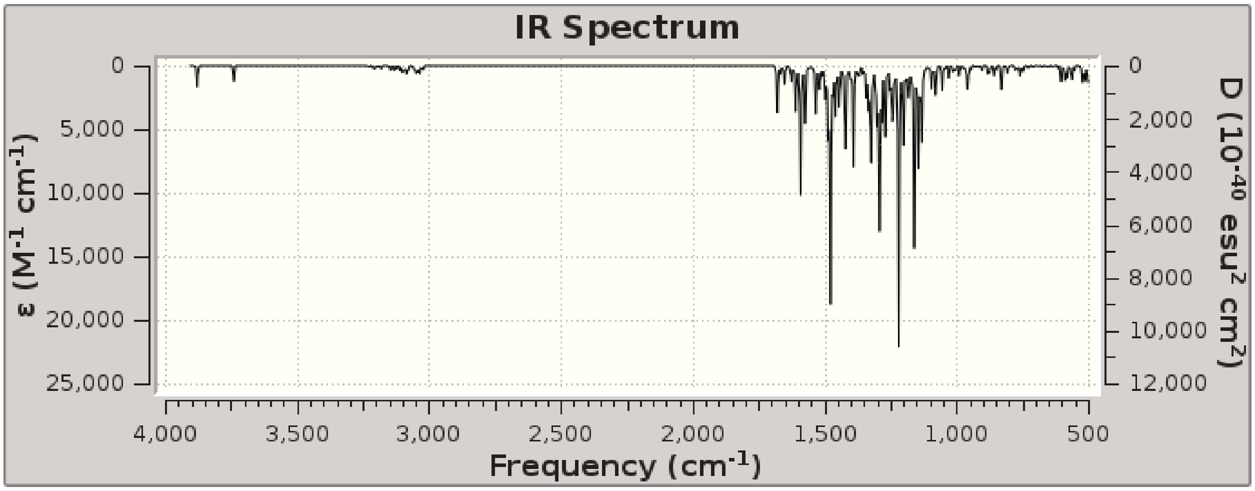

Figure S23. Calculated UV-Vis spectrum for probe **B + Zn(OH$_2$)$^{2+}$.**

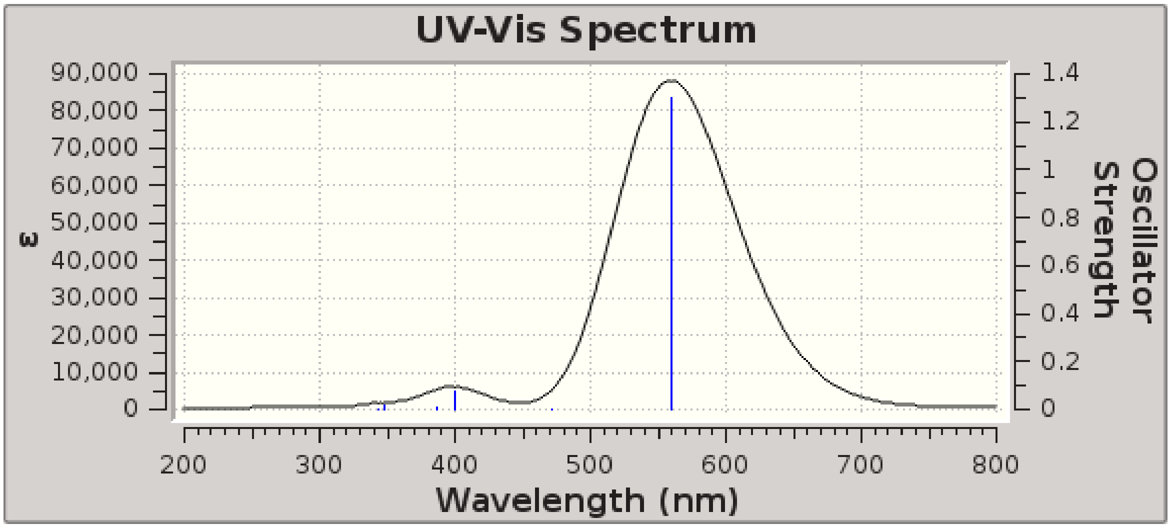

Figure S24. Calculated UV-Vis spectrum for probe **B + Zn(OH$_2$)$^{2+}$.**

Table S6. Excitation energies and oscillator strengths listing for probe **B + Zn(OH$_2$)$^{2+}$**.

**Excited State**	**Nature**	**E (eV)**	**λ (nm)**	***f***	**Orbital transitions**	**Normalized coefficient**
1	Singlet-A	2.2142	559.96	1.3007	185 → 186	0.70537
2	Singlet-A	2.6276	471.85	0.0002	185 → 187	0.70345
3	Singlet-A	3.0961	400.45	0.0763	183 → 186<br>184 → 186<br>185 -> 188	0.14237<br>0.66917<br>-0.12017
4	Singlet-A	3.2110	386.13	0.0088	184 → 186<br>185 → 188	0.11209<br>0.68637

5	Singlet-A	3.5704	347.26	0.0143	183 → 186<br>184 → 187<br>185 → 189<br>185 → 190<br>185 → 191	0.49214<br>0.10203<br>-0.27024<br>0.34722<br>-0.15623
6	Singlet-A	3.6060	343.82	0.0034	183 → 186<br>184 → 187	-0.11648<br>0.67675

Theoretical Data for probe **B + $Zn(OH_2)_2^{2+}$.**

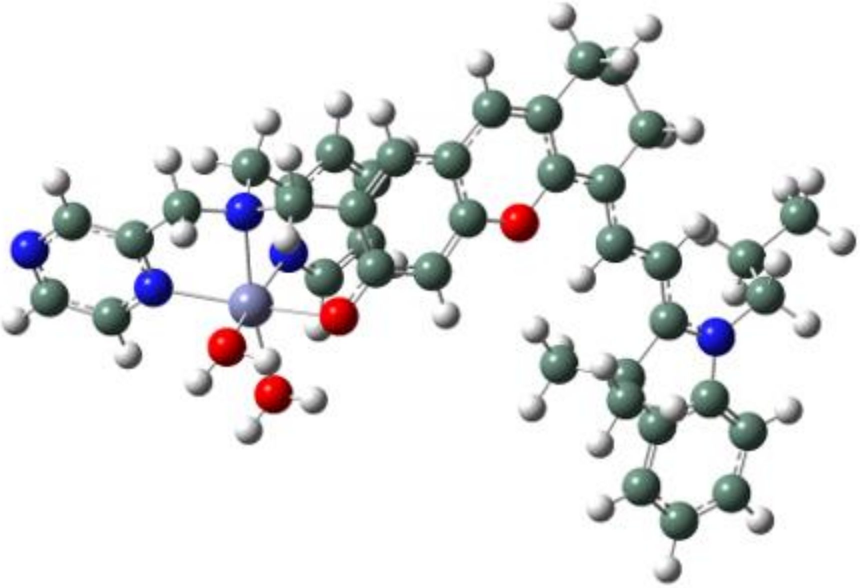

Figure S25. GaussView representation of probe **B + $Zn(OH_2)_2^{2+}$.**

znb3		
/home/rluck/calculation/liu/mingxi/superior/znb3....		
File Type	.chk	
Calculation Type	FREQ	
Calculation Method	RAPFD	
Basis Set	6-311+G(2D,P)	
Charge	2	
Spin	Singlet	
Solvation	scrf=solvent=water	
Electronic Energy	-3903.916112	Hartree
RMS Gradient Norm	0.000004	Hartree/Bohr
Imaginary Freq	0	
Dipole Moment	12.410141	Debye
Polarizability (α)	986.301767	a.u.
Point Group		

Figure S26. Illustration of the computational results for probe **B + $Zn(OH_2)_2^{2+}$.**

Table S7. Computational results for probe **B + $Zn(OH_2)_2^{2+}$.**

Row	Symbol	X	Y	Z
1	C	-5.81071	-4.0715	-0.58159
2	C	-6.99603	-4.78582	-0.39896
3	C	-8.17557	-4.12479	-0.06817
4	C	-8.20953	-2.7404	0.084604
5	C	-7.0212	-2.05548	-0.10981
6	C	-5.83426	-2.70046	-0.43027
7	N	-6.78134	-0.67929	-0.02848
8	C	-5.48394	-0.38952	-0.24128
9	C	-4.73181	-1.68002	-0.55047
10	C	-7.81287	0.27784	0.324851
11	C	-7.89985	0.502236	1.82949
12	C	-8.97841	1.519104	2.172484
13	C	-3.64041	-1.96538	0.491719
14	C	-4.16721	-1.68122	-1.97882
15	C	-4.98276	0.899973	-0.1542
16	C	-3.65533	1.236275	-0.39236
17	C	-3.10595	2.505361	-0.27678
18	C	-3.91612	3.697823	0.163945
19	C	-3.05222	4.78897	0.786711
20	C	-1.90492	5.158839	-0.14444

Row	Symbol	X	Y	Z
21	C	-1.09159	3.947856	-0.49598
22	C	-1.74657	2.694993	-0.56959
23	C	0.254816	3.996228	-0.73748
24	C	0.986455	2.837373	-1.06858
25	C	0.280583	1.62512	-1.17306
26	O	-1.05311	1.597314	-0.93255
27	C	2.379098	2.788981	-1.24086
28	C	3.02838	1.604968	-1.48394
29	C	2.28525	0.383867	-1.6288
30	C	0.889253	0.43151	-1.48004
31	O	2.89803	-0.74118	-1.82567
32	C	4.521831	1.530175	-1.48228
33	N	5.011191	0.773902	-0.30444
34	C	6.91555	-0.49413	0.542833
35	C	3.181047	0.777282	1.356648
36	C	2.200357	1.56072	1.946605
37	C	0.998933	0.973664	2.307767
38	C	0.813093	-0.3835	2.079991
39	C	1.837785	-1.09604	1.485247
40	N	2.994237	-0.52815	1.132512
41	C	8.142448	-0.50018	1.196968
42	N	8.552149	-1.53064	1.925101
43	C	7.729098	-2.57081	2.017291
44	C	6.495739	-2.58202	1.38491
45	N	6.101355	-1.54495	0.650423
46	Zn	4.266363	-1.27188	-0.37651
47	O	5.458771	-1.63496	-2.22011
48	C	6.467955	0.655009	-0.31676
49	C	4.523559	1.336702	0.965257
50	H	-4.89097	-4.58914	-0.8349
51	H	-6.99769	-5.86449	-0.51169
52	H	-9.08736	-4.69434	0.077005
53	H	-9.13088	-2.23603	0.350995
54	H	-7.6109	1.211916	-0.20232
55	H	-8.75936	-0.09816	-0.06825
56	H	-8.10492	-0.45462	2.320805
57	H	-6.92497	0.839938	2.19634
58	H	-9.04188	1.675237	3.251534
59	H	-8.77064	2.486804	1.706675
60	H	-9.96084	1.186437	1.825109
61	H	-3.21793	-2.95573	0.308525
62	H	-2.8304	-1.23713	0.443114

Row	Symbol	X	Y	Z
63	H	-4.05503	-1.95156	1.501719
64	H	-4.95192	-1.46569	-2.70666
65	H	-3.37158	-0.94619	-2.10341
66	H	-3.75492	-2.66771	-2.20157
67	H	-5.67131	1.688082	0.125142
68	H	-2.97949	0.449231	-0.6978
69	H	-4.4671	4.106757	-0.69328
70	H	-4.67122	3.374781	0.88581
71	H	-3.66129	5.669236	1.007132
72	H	-2.64442	4.43172	1.739167
73	H	-2.3106	5.598428	-1.06483
74	H	-1.25612	5.915231	0.30445
75	H	0.780218	4.944211	-0.66035
76	H	2.948471	3.706806	-1.12411
77	H	0.310157	-0.48268	-1.53629
78	H	4.954295	2.537681	-1.48264
79	H	4.886271	1.001124	-2.36581
80	H	2.373703	2.619892	2.095712
81	H	0.210388	1.571363	2.751977
82	H	-0.11338	-0.88032	2.340835
83	H	1.73983	-2.15213	1.261533
84	H	5.941645	-2.45719	-2.36077
85	H	4.790933	-1.58455	-2.916
86	H	6.961697	1.5808	-0.00061
87	H	6.77954	0.452301	-1.34535
88	H	5.237875	1.07045	1.750328
89	H	4.486692	2.429652	0.924295
90	O	3.242187	-3.13214	-0.57821
91	H	2.659184	-2.87768	-1.30953
92	H	3.718333	-3.91967	-0.86617
93	H	8.803352	0.358195	1.126402
94	H	5.818395	-3.42359	1.468732
95	H	8.051229	-3.41725	2.613948

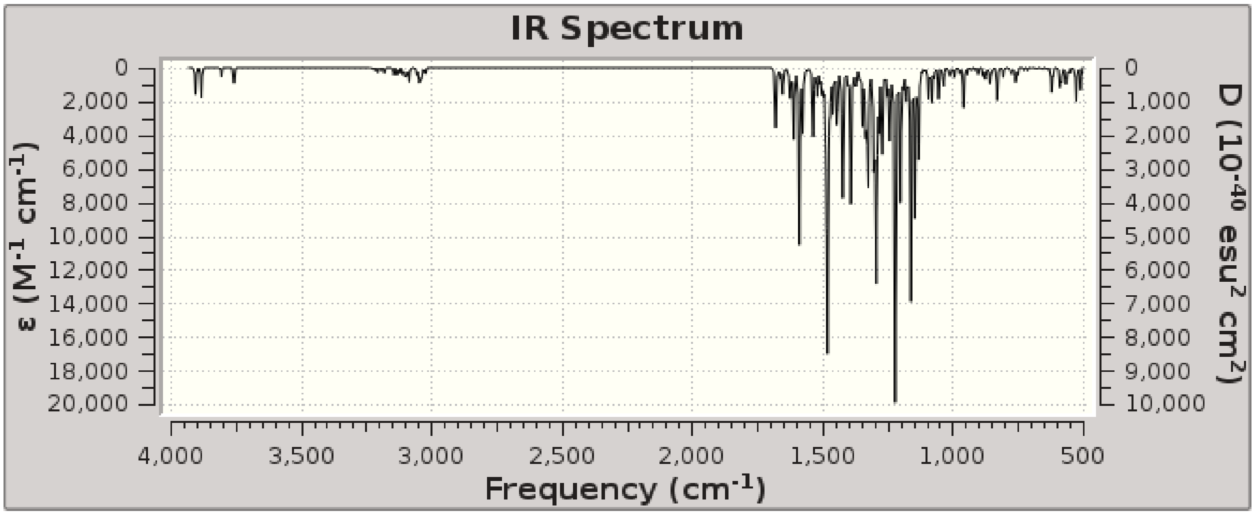

Figure S27. Calculated IR spectrum for probe **B + $Zn(OH_2)_2^{2+}$.**

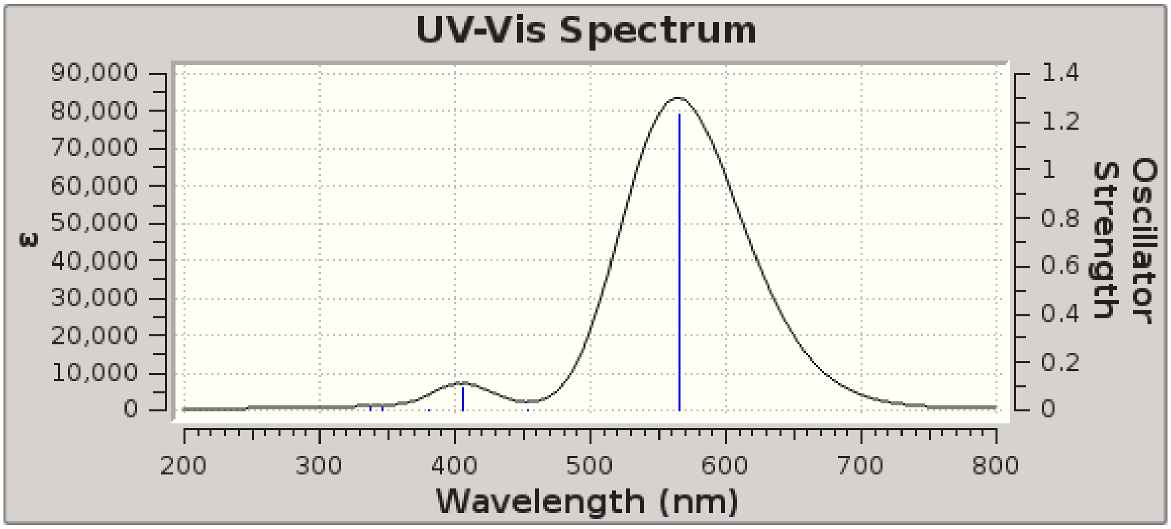

Figure S28. Calculated UV-Vis spectrum for probe **B + $Zn(OH_2)_2^{2+}$.**

Table S8. Excitation energies and oscillator strengths listing for probe **B + $Zn(OH_2)_2^{2+}$.**

Excited State	Nature	E (eV)	λ⚜(nm)	*f*	Orbital transitions	Normalized coefficient
1	Singlet-A	2.1942	565.05	1.2313	190 → 191	0.70479
2	Singlet-A	2.7348	453.35	0.0014	190 → 192	0.70339
3	Singlet-A	3.0550	405.84	0.0966	188 → 191<br>189 → 191	-0.13454<br>0.67953
4	Singlet-A	3.2522	381.24	0.0039	190 → 193	0.69421
5	Singlet-A	3.5775	346.57	0.0084	188 → 191<br>190 → 194<br>190 → 195<br>190 → 196	0.58754<br>0.13966<br>-0.29918<br>0.11616
6	Singlet-A	3.6727	337.58	0.0048	188 → 191<br>189 → 192<br>190 → 194<br>190 → 196	-0.16423<br>-0.31328<br>0.54901<br>0.23397

Table S9. Calculated electronic transitions (nm) and corresponding oscillator strengths (f) and their percentage contribution to the UV/Vis spectra.

Species	*Transition (HOMO-1/LUMO)*	*Wavelength (nm)*	*f*	%
Intermediate 4	S0-S2<br>165 → 167	699	1.4954	99.9
Intermediate 9	S0-S2<br>165 → 167	701	1.4907	99.8
probe A	S0-S2<br>176 → 178	693	1.1326	78.9
probe B	S0-S2<br>176 → 178	699	0.7194	99.9
Zinc Complex 4	*S0-S1 (HOMO/LUMO)*<br>180 ->181	744	1.3627	99.9
Zinc Complex 9	S0-S2<br>179 → 181	702	1.3926	99.6
Zinc Complex 4 OH^-	*S0-S1 (HOMO/LUMO)*<br>185 → 186	765	1.2967	99.0
Zinc Complex 9 OH^-	S0-S2<br>185 → 187	711	1.3857	99.8

Table S10. Drawings of the HOMO-1/LUMO states for Intermediates **4** and **9**, and for probes **A** and **B**.

	Intermediate 4	*Intermediate 9*	*probe A*	*probe B*
LUMO	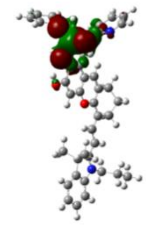 (a)	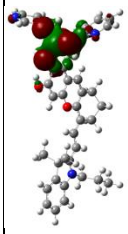 (c)	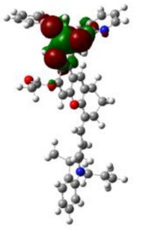 (e)	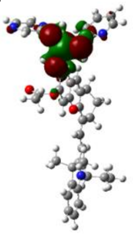 (g)
HOMO-1	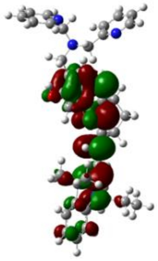 (b)	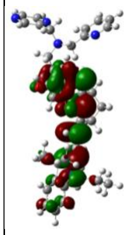 (d)	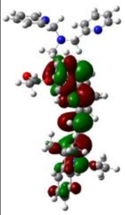 (f)	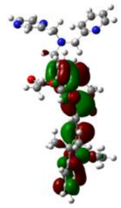 (h)

Table S11. Drawings of the HOMO-1/LUMO distributions for the Zn complexes explored in this work. Note that exceptions are observed for (b) and (f), corresponding to Zn complex **4**, for which the excitation is predicted as an HOMO/LUMO transition.

	Zinc complex 4	*Zinc complex 9*	*Zinc complex 4 with OH$^-$*	*Zinc Complex 9 with OH$^-$*
LUMO	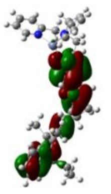 (a)	(c) 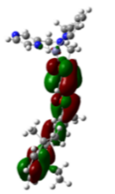	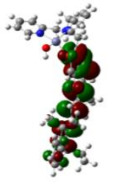 (e)	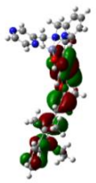 (g)

HOMO-1	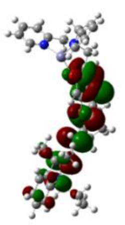 (b) **HOMO**	(d) 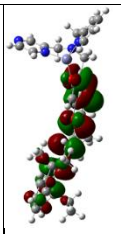 **HOMO-1**	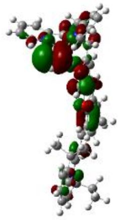 (f) **HOMO**	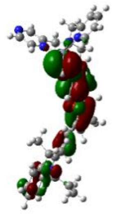 (h) **HOMO-1**

Table S12. Calculated electronic transitions (nm) and corresponding oscillator strengths (f) and their percentage contribution to the UV/Vis spectra for the $[Zn(OH_2)]^{2+}$ and $[Zn(OH_2)_2]^{2+}$ moieties attached to probes **A** and **B**.

**Complex**	**Excited State 1**	**Wavelength (nm)**	***f***	**%**
**A** $Zn(OH_2)^{2+}$	185→186	560.9	1.3017	99.5
**A** $Zn(OH_2)_2^{2+}$	190→191	566.0	1.2351	99.4
**B** $Zn(OH_2)^{2+}$	185→186	560.0	1.3007	99.5
**B** $Zn(OH_2)_2^{2+}$	190→191	565.1	1.2313	99.3

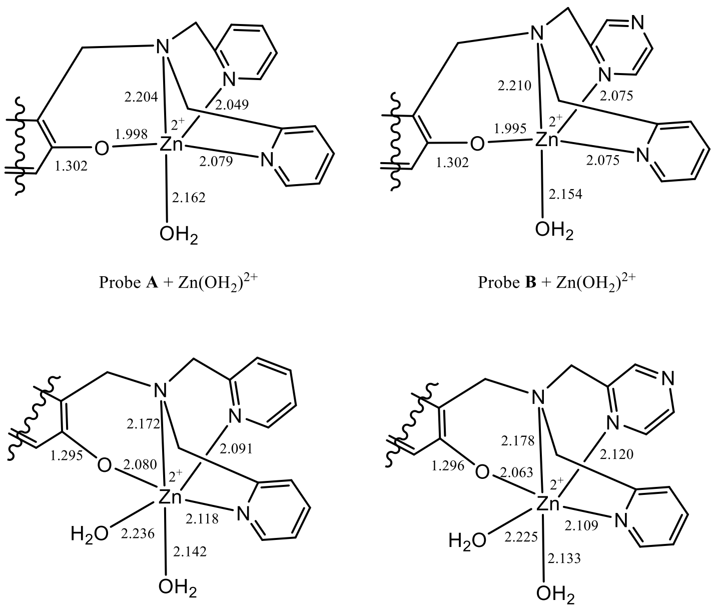

Figure S29. Calculated coordination bond distances (Å) for probes **A** and **B** with $Zn(OH_2)^{2+}$ and $Zn(OH_2)_2^{2+}$.

10. Comparison of Zn concentration via atomic absorption spectroscopy and fluorescence change

The ability of the fluorescent probe A to monitor the concentration of free Zn^{2+} ions in solution was assessed by preparing standard solutions of a known Zn^{2+} concentration and then also measuring the concentration through fluorescence measurement with the probe. As a result, 93.1% and 99.5% accuracy was demonstrated as shown in Fig. S30.

Two stock solutions ($ZnSO_4$, 0.0595 mg/L and 0.0424 mg/L) were prepared and tested.

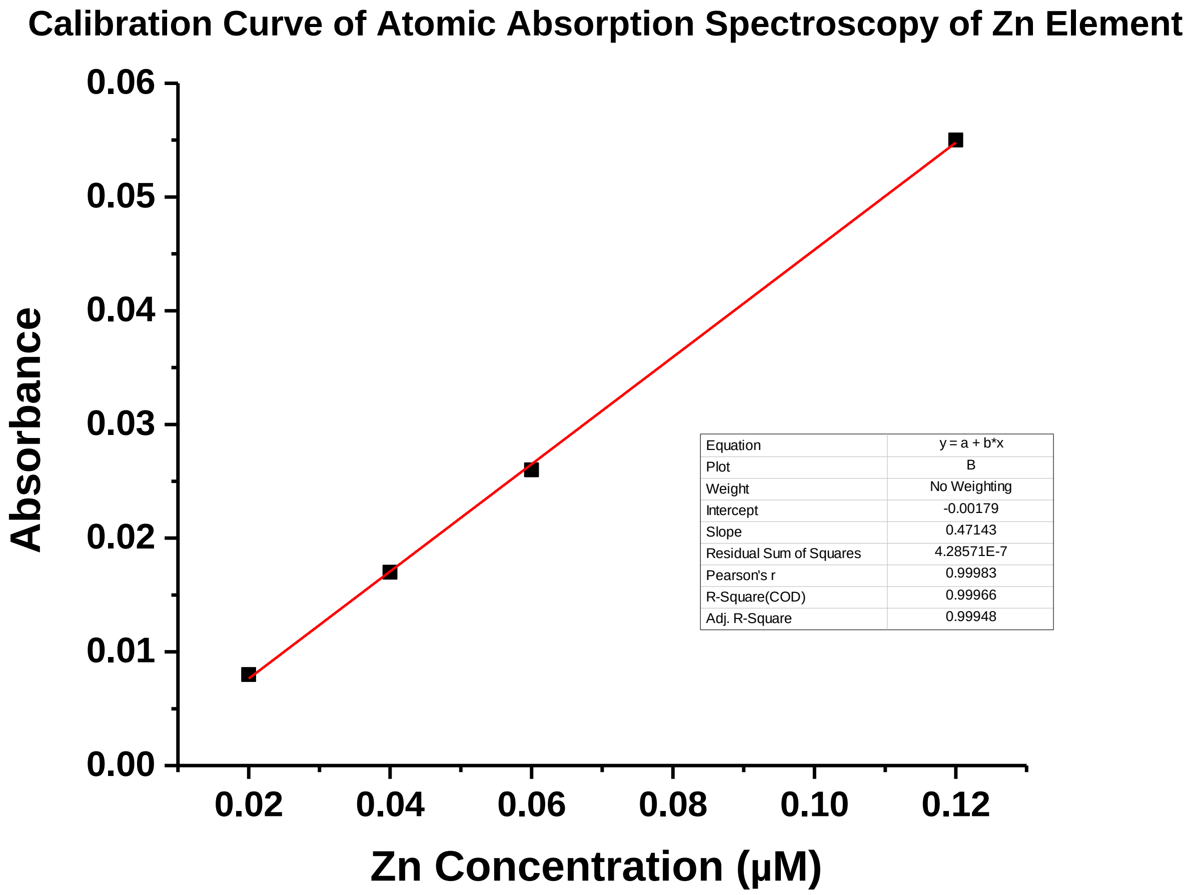

Figure S30. Calibration curve of atomic absorption spectroscopy of Zn element.

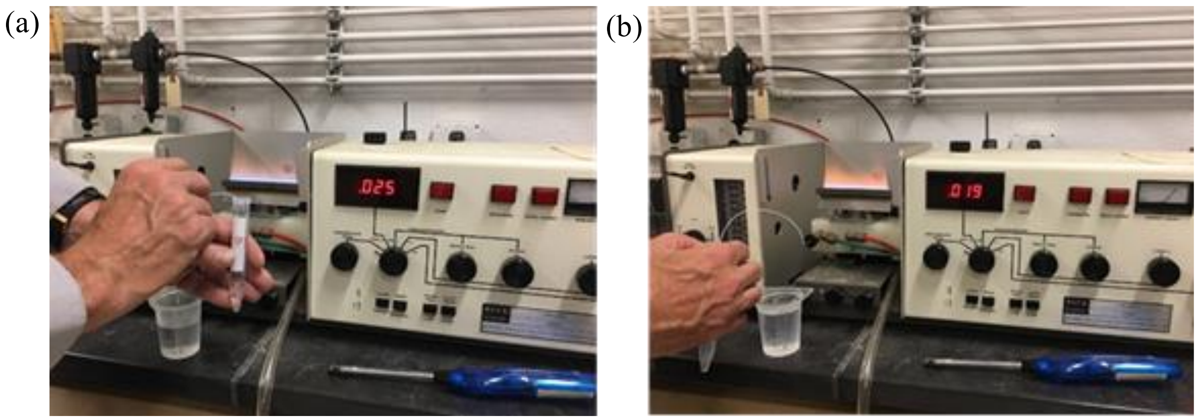

Figure S31. Absorption of stock solution samples obtained in atomic absorption spectroscopy and calibration curve; **(a)** Zn^{2+} concentration of sample **1** was calculated to be 0.06mg/L= 0.923μM, **(b)** sample **2** was calculated to be 0.0438mg/L= 0.673μM.

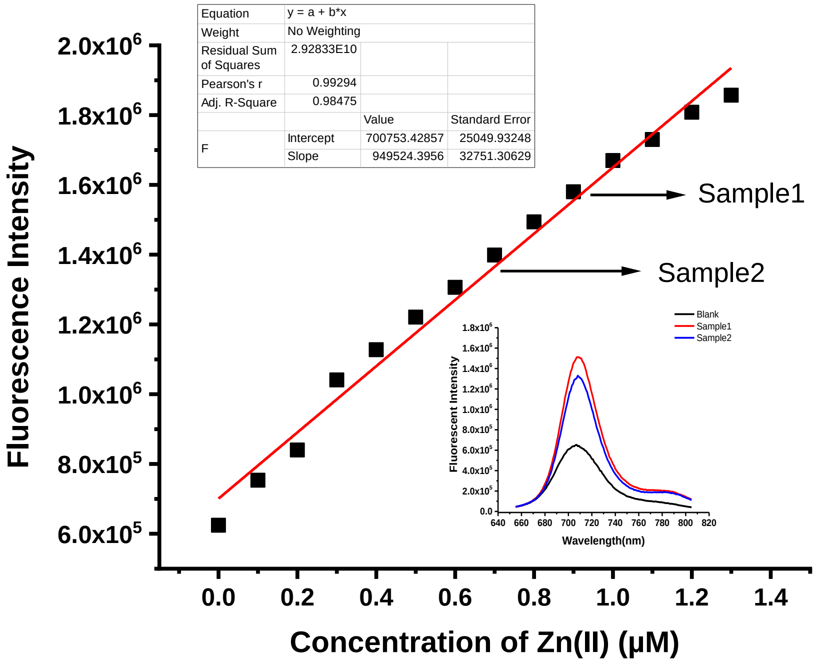

Figure S32. Determination of Zn^{2+} concentration according to fluorescence changes.

In order to determine the Zn concentration of the stock solution, samples were added into the solution of fluorescent probe A (5μM) to measure fluorescent changes.

For sample **1**, Concentration of Zn is calculated to be 0.852 uM, accuracy is 93.1%.

For sample **2**, Concentration of Zn is calculated to be 0.655 uM, accuracy is 99.5%.

11. References

1. Goswami, S., et al., *A new rhodamine based colorimetric 'off-on' fluorescence sensor selective for Pd2+ along with the first bound X-ray crystal structure.* Chem Commun (Camb), 2011. **47**(32): p. 9101-3.
2. Zhang, S., et al., *Near-Infrared Fluorescent probes with Large Stokes Shifts for Sensing Zn(II) Ions in Living Cells.* ACS Sens, 2016. **1**(12): p. 1408-1415.
3. Yuan, L., et al., *A unique class of near-infrared functional fluorescent dyes with carboxylic-acid-modulated fluorescence ON/OFF switching: rational design, synthesis, optical properties, theoretical calculations, and applications for fluorescence imaging in living animals.* J Am Chem Soc, 2012. **134**(2): p. 1200-11.
